# Whole exome sequencing reveals mutations in *FAT1* tumor suppressor gene clinically impacting on peripheral T-cell lymphoma not otherwise specified

**DOI:** 10.1038/s41379-019-0279-8

**Published:** 2019-04-25

**Authors:** Maria Antonella Laginestra, Luciano Cascione, Giovanna Motta, Fabio Fuligni, Claudio Agostinelli, Maura Rossi, Maria Rosaria Sapienza, Simona Righi, Alessandro Broccoli, Valentina Indio, Federica Melle, Valentina Tabanelli, Angelica Calleri, Domenico Novero, Fabio Facchetti, Giorgio Inghirami, Elena Sabattini, Francesco Bertoni, Stefano A. Pileri

**Affiliations:** 1 Department of Experimental, Diagnostic, and Specialty Medicine, University of Bologna, Bologna, Italy; 2 Università della Svizzera Italiana, Institute of Oncology Research, Bellinzona, Switzerland; 3 Division of Haematopathology, IEO European Institute of Oncology IRCCS, Milan, Italy; 4 Department of Genetics and Genome Biology, The Hospital for Sick Children, Toronto, Canada; 5 Division of Cancer Research Center “Giorgio Prodi” University of Bologna, Bologna, Italy; 6 Division of Pathological Anatomy, Quality and Safety of Diagnosis and Treatment, Città della Salute e della Scienza, Turin, Italy; 7 Division of Pathology Department of Molecular and Translational Medicine, Section of Pathology, University of Brescia, Brescia, Italy; 8 Department of Pathology and Laboratory Medicine, Weill Cornell Medical College, New York, NY USA

**Keywords:** T-cell lymphoma, Next-generation sequencing

## Abstract

Peripheral T-cell lymphoma not otherwise specified represents a diagnostic category comprising clinically, histologically, and molecularly heterogeneous neoplasms that are poorly understood. The genetic landscape of peripheral T-cell lymphoma not otherwise specified remains largely undefined, only a few sequencing studies having been conducted so far. In order to improve our understanding of the genetics of this neoplasm, we performed whole exome sequencing along with RNA-sequencing in a discovery set of 21 cases. According to whole exome sequencing results and mutations previously reported in other peripheral T-cell lymphomas, 137 genes were sequenced by a targeted deep approach in 71 tumor samples. In addition to epigenetic modifiers implicated in all subtypes of T-cell neoplasm (*TET2, DNMT3A, KMT2D, KMT2C, SETD2*), recurrent mutations of the *FAT1* tumor suppressor gene were for the first time recorded in 39% of cases. Mutations of the tumor suppressor genes *LATS1, STK3, ATM, TP53*, and *TP63* were also observed, although at a lower frequency. Patients with *FAT1* mutations showed inferior overall survival compared to those with wild-type *FAT1*. Although peripheral T-cell lymphoma not otherwise specified remains a broad category also on molecular grounds, the present study highlights that *FAT1* mutations occur in a significant proportion of cases, being provided with both pathogenetic and prognostic impact.

## Introduction

Peripheral T-cell lymphoma not otherwise specified represents the commonest entity among nodal peripheral T-cell lymphomas [1]. Peripheral T-cell lymphoma not otherwise specified is a kind of Pandora’s box, corresponding to a group of peripheral T-cell lymphomas that cannot be classified into one of the well-defined disease categories due to its extreme cytological, phenotypic, and molecular heterogeneity [1]. Gene expression and microRNA profiling studies have improved peripheral T-cell lymphoma not otherwise specified diagnostic accuracy and pathogenetic understanding [2, 3]. Two main subgroups have been identified, characterized by high expression of either GATA3 or TBX21/T-bet transcription factors and downstream target genes [4].

The next generation sequencing approach has recently led to the discovery of recurrent somatic mutations of genes involved in the epigenetic regulation (*KMT2D, TET2, KDM6A, DNMT3A, CREBBP, KMT2A*), signaling pathways (*TNFAIP3, APC, CHD8, ZAP70, NF1, TNFRSF14, TRAF3*), and tumor suppression (*TP53, FOXO1, BCORL1, ATM*) [5–7].

Most studies have been performed by targeted sequencing with the limitation of a gene-discovery analysis based on a restricted list of candidate genes [5, 6]; only a few peripheral T-cell lymphomas not otherwise specified have been analyzed by whole exome sequencing [7].

In this study, we performed whole exome sequencing in a discovery set of 21 samples of peripheral T-cell lymphoma not otherwise specified alongside with RNA-sequencing. This was followed by targeted sequencing, to better define the genetic landscape, identify novel recurrent gene mutations, and improve our understanding of common genetic lesions, which might functionally contribute to tumorigenesis.

## Material and methods

### Case collection

The study comprised 71 peripheral T-cell lymphoma not otherwise specified samples divided into a discovery panel obtained from frozen lymph node biopsies (*N* = 21) and an extension panel obtained from formalin-fixed paraffin embedded tissue samples (*N* = 50). Matched normal DNA was obtained from saliva in 12 patients (four included in the discovery set and eight in the extension panel). Further 20 saliva samples of healthy volunteers were sequenced to create an internal normal control (PoN). In addition, eight reactive formalin-fixed paraffin embedded lymph nodes with paracortical hyperplasia were retrieved for targeted resequencing. In all tumor cases, the fraction of neoplastic cells was estimated to be >75% by morphology and immunohistochemistry. All the cases had been diagnosed by expert hematopathologists (E.S., C.A., and S.A.P.) according to the revised 4th edition of the WHO Classification of Tumor of Haematopoietic and Lymphoid Tissues and collected at diagnosis before any treatment. Peripheral T-cell lymphomas with T follicular helper phenotype were not included in the present cohort, since they now belong to the new category of angioimmunoblastic T-cell lymphoma and other nodal lymphomas of T follicular helper origin. In addition, the signature reported by Iqbal et al. [4] for the subclassification of peripheral T-cell lymphomas not otherwise specified, was applied to the 18 cases of the discovery panel, which underwent both whole exome and RNA-sequencing. In those of the extension panel, GATA3 and TBX21/T-bet were searched by immunohistochemistry.

Written informed consent was obtained from all patients according to the principles of the Helsinki declaration after approval of the Internal review Board (Prot. Numb. 001-2011-U-Tess).

Patients characteristics are summarized in Supplementary Table S1.

### Whole exome sequencing

Genomic DNA from tumor samples was extracted using the semiautomatic extractor MagCore nucleic acid extractor with the MagCore Genomic DNA Tissue Kit (RBC Bioscience Corp, Taiwan); DNA from saliva samples was collected using the Oragene-DNA collection kit and extracted according to the manufacturer’s protocol (DNA Genotek Inc., ON, Canada). DNA was quantified by the Quant-it PicoGreen dsDNA Assay Kit (Invitrogen Life technologies, UK) according to the manufacturer’s protocol. One microgram of DNA was sheared using an M220 Ultrasonicator (Covaris) into 100–500 bp fragments and quality control of fragmentation was assessed using a DNA-7500 kit (Agilent, USA). We performed paired-end library using pre-enrichment DNA libraries preparation (TruSeq DNA Sample Preparation v2) according to the manufacturer’s protocol (Illumina, San Diego, USA). Briefly, after end repair, adenylate 3′ ends and ligate adapters steps, we performed a PCR reaction to selectively enrich those DNA fragments that had adapter molecules on both ends. PCR libraries products were purified by AmpureXP beads (Beckman-Coulter, CA, USA) and exome enrichment capture was performed according to Illumina TruSeq Exome Enrichment protocol (Illumina, San Diego, USA). Two 20-h biotinylated bait-based hybridizations were performed, each followed by streptavidin magnetic beads binding, a washing step and an elution step. A 10-cycle PCR enrichment was performed after the second elution and the enriched libraries were subjected to quality control analysis using a DNA-1000 kit (Agilent, USA). The quantification was performed by the Quant-it PicoGreen dsDNA Assay Kit according to manufacturer’s protocol (Invitrogen, Life Technologies, USA).

The paired-end libraries (2 × 100 base pair) were sequenced on an Illumina HiScan SQ (Illumina, San Diego, USA) following the manufacturer’s instructions, generating an average of about 60 million 100 bp paired-ends raw reads per sample, with coverage calculated on hg19 RefSeq nonredundant exome length, ranging from 35× to 108×. Quality control on raw reads was performed using FastQC V0.10.0 (http://www.bioinformatics.babraham.ac.uk/projects/fastqc/).

### Whole exome sequencing analysis

Paired-end reads were aligned to human reference sequence GRCh37 using the Burrows–Wheeler Aligner (BWA version 0.6.1) [8]. Multiple mapped reads pairs with identical external coordinates were collapsed to remove potential PCR duplicates using SAM tools command [9]. Mapping quality score recalibration and local realignment around insertions and deletions (indels) was performed using the Genome Analysis Toolkit (GATK). Single nucleotide variants and small indels were called separately using the GATK Unified-Genotyper [10]. Germline mutations present in saliva from unaffected individuals were excluded.

Annovar tool (http://www.openbioinformatics.org/annovar/) [11] was used for functional annotation of variants, exonic functions and nonsynonymous variants, including stop-gain single nucleotide variants, splicing, and frameshift indels. All the mutations found were manually checked and explored using the Integrative Genomic Viewer 2.03 [12].

### Candidate somatic variants

The final list of putative somatic variants was identified using the following filtering constrains. Variants with total depth in tumor and internal normal control lower than 10× were filtered out. Then, we first selected somatic variants with frequency >10% in tumor samples and less than 3% in normal samples. In samples without a matched normal control, we selected variants with depth >20× and frequency >25%. To remove systematic errors, we created an internal database with all the variants present in normal samples and excluded all variants that were found to be present in any of the normal samples. Known germline variants reported at dbSNP137 and the 1000 Genomes Project [13] were excluded, retaining only those variants that were previously reported in the COSMIC database [14]. Since 18 discovery samples underwent both whole exome sequencing and RNA-sequencing, variants were selected according to the correspondence between whole exome and RNA-sequencing. The role of these genes was manually curated in pathways. To eliminate ambiguous mapping from captured pseudogenes, each variant with a flanking 20-base context sequence around its genomic position was mapped to the hg19 reference genome using the BLAT algorithm [7]. Based on these filtering steps, 92 genes emerged from the whole exome analysis. Further 45 genes previously reported as mutated in peripheral T-cell lymphomas but not recorded in the discovery set, were added to them to generate a 137 genes panel, which was used for MiSeq validation by resequencing of all exons.

### Whole transcriptome sequencing

Total RNA from 18 samples belonging to the discovery set was extracted with Trizol according to manufacturer’s instructions (Invitrogen, Life Technologies). RNA was quantified using ND-1000 spectrophotometer running software version 3.0.1 (NanoDrop Technologies, Inc., Rockland, DE). Paired-end libraries (2 × 75 base pair) were prepared according to the TruSeq RNA sample preparation v2 protocol (Illumina, San Diego, USA). Two micrograms of Poly(A) +RNA were purified from total RNA using poly-T oligo attached magnetic beads and then used for fragmentation into 130–290 bp fragments. First strand of cDNA synthesis was performed using reverse transcriptase enzyme (SuperScript II, Invitrogen, Life Technologies, USA) and random hexamer primer, followed by generation of double-stranded (ds) cDNA. AmpureXP beads (Beckman- Coulter, Brea CA) were used to purify the ds cDNA and end repair step was performed to convert the overhangs, resulting from fragmentation, into blunt ends by 3′–5′ exonuclease activity. A single “A” nucleotide was added to the 3′ ends of the blunt fragments to prevent them from ligating to one another during the adapter ligation reaction. This approach was adopted to ensure a low rate of chimera (concatenated template) formation. Subsequently, sequencing adapters were added to the ends of the ds cDNA fragment and a PCR reaction was used to selectively enrich those ds cDNA fragments that had adapter molecules on both ends, amplifying the amount of ds cDNA in the final libraries. Lastly, PCR libraries products were purified by AmpureXP beads and quality control analysis was assessed using a DNA-1000 (Agilent, USA). The quantification was performed by the Quant-it PicoGreen dsDNA Assay Kit according to the manufacturer’s protocol (Invitrogen, Life Technologies, USA). The resulting libraries were sequenced on an Illumina HiScan SQ (Illumina, San Diego, USA) following the manufacturer’s instructions.

### Whole transcriptome sequencing analysis

Transcriptome data analysis was performed according to the criteria reported by Abate et al. [15]. The results of RNA-sequencing analysis were used to identify differentially expressed genes by the limma package (Bioconductor) [16]. Gene set enrichment analysis (GSEA) was performed using GSEA software and Molecular Signature Database (MSigDB) [17] on ranked gene list based on base-2 fold change logarithm transformation.

### Targeted sequencing of selected genes

We performed targeted sequencing of 71 peripheral T-cell lymphoma not otherwise specified samples (including the 21 which had undergone whole exome sequencing), 12 normal saliva samples, and 8 hyperplastic lymph nodes. DNA was extracted using the semiautomatic MagCore nucleic acid extractor with the MagCore Genomic DNA Tissue Kit (RBC Bioscience Corp, Taiwan) and quantified using the Quant-iT PicoGreen dsDNA Assay Kit (Invitrogen Life technologies, UK) according to the manufacturer’s protocol. We performed quality check for the 58 formalin-fixed paraffin embedded tissue samples (50 PTCLs-NOS and 8 hyperplastic nodes) using the KAPA Human Genomic DNA Quantification and QC Kit to verify whether the DNA quality of each sample was adequate for successful library preparation. We designed a six custom amplicon panel to specifically sequence all 137 coding DNA sequence of genes with DesignStudio, an online software, available at Illumina website (https://designstudio.illumina.com/). We used the TruSeq Custom Amplicon Kit with 250 ng of DNA per sample and the amplicon libraries were loaded on MiSeq instrument (Illumina, Inc., San Diego, CA, USA) to generate 2 × 75-bp paired reads, according to the manufacturer’s instructions. All libraries passed the quality check and the MiSeq targeted sequencing approach allowed us to reach a median coverage depth of 1000×.

### Targeted sequencing analysis

Raw fastq files were preprocessed to discard sequences with a Phred-quality score of 30 and below (threshold: 70% of reads were above Q30). Fastq files were demultiplexed with CASAVA and raw reads were aligned using BWA [8]. Variant detection was performed with VarScan2 [18] using the following criteria: allowable ambiguous alignments, at least 90% of a read having to match the reference genome, hiding the unmatched ends of reads, with at least 10% variant allelic fraction, and at least 3 variant reads to call a variant.

After annotation, the information from different databases, 1000 Genomes Project, dbSNP and the Exome Variant Server, was exploited to reduce the number of candidate variants. Variant filtering was performed using the following criteria: exclude variants with an allele prevalence >1% in the 1000 Genomes Project, considering only those variants reported in the COSMIC database [14] and filter out common variants identified in previous exome analysis, nonpathogenic variants reported in dbSNP and low-quality calls. The survived variants underwent manual curation and variant priorization through visual inspection of alignments. Synonymous variants and intronic variants >2 bp from the coding sequence were excluded. Variants were also manually cross-referenced with the COSMIC database [14] and cBioPortal [19, 20] as well as variants present in the normal samples and hyperplastic lymph nodes were discarded from the analysis.

### Prediction of single point mutations on protein stability

The INPS-MD tool (Impact of non-synonymous mutations on protein stability-multi dimension), which computes the ΔΔG values of protein variant, was used to predict and annotate the effect of single nonsynonymous mutations on the protein stability from its sequence. INPS is based on support vector regression and is trained to predict the thermodynamic free energy change upon single-point variations in protein sequences (http://inps.biocomp.unibo.it) [21].

### Immunohistochemistry

Immunohistochemistry was performed on 2 μm-thick dewaxed sections after antigen retrieval (PTLink at 92 °C for 5 min in EnVision Flex Target Retrieval Solution High pH) using a Dako AutoStainer Link48 (Dako Agilent, Glostrup, Denmark). Sections were stained for: GATA3 (clone EPR16651 Abcam), TBX21/T-bet (clone 4B10, 4BD Pharmingen), CD2 (clone AB75, Leica), CD3 (polyclonal, Agilent), CD4 (clone 1F6, Leica), CD5 (clone 54/F6, Agilent), CD7 (clone 580, Leica), CD8 (clone 144B, Agilent), CD30 (clone Ber-H2, Agilent), PD1 (clone NAT1, CNIO), BCL6 (clone PG-B6P, Agilent), CD10 (clone 56C6, Leica), CXCL13 (polyclonal, R&D Systems), TIA1 (clone 249, Immunotech), Granzyme B (clone 11F1, Leica), Perforin (clone 5B10, Bio Optical), CD20 (clone L26, Agilent), PAX5 (clone DAK-PAX5, Agilent), CD21 (clone 1F8, Agilent), and Ki-67 (clone Mib1, Agilent). Alkaline phosphatase anti-alkaline phosphatase was applied for antibody detection.

Immunohistochemical results were evaluated with a semiquantitative approach (H-score, range 0–300) multiplying the percentage of positive cells (0–100%) and the intensity of staining (0–3+).

### Survival analysis

Clinical information and complete follow-up were available for 61/71 cases for which MiSeq targeted sequencing was performed. All the cases were staged III–IV. On therapeutic grounds, quite different schedules had been used, this reflecting the case collection that spanned over 10 years. Overall survival (OS) was calculated from the time of diagnosis to death or last follow-up. Statistical analyses were carried out by the CRAN survival package (version 2.42-3) using R (v3.2.1) [22]. Survival data were analyzed with the Kaplan–Meier estimator method. The limit of significance for all analyses was defined as *P* < 0.05 for the log-rank Mantle–Cox test.

## Results

### Identification of peripheral T-cell lymphoma not otherwise specified coding mutations by whole exome sequencing

To discover novel recurrent mutations in peripheral T-cell lymphoma not otherwise specified, we performed whole exome sequencing of 21 frozen tumor samples.

Tumor samples were selected based on a high percentage of cancer cells (≥75%) to minimize contamination with normal cells that can hide the identification of somatic mutation. We achieved a 40-fold mean sequence coverage of exonic regions (range, 15×−55×) and 74% of exome was covered at least 10× (range 57–76%).

**Fig. 1:**
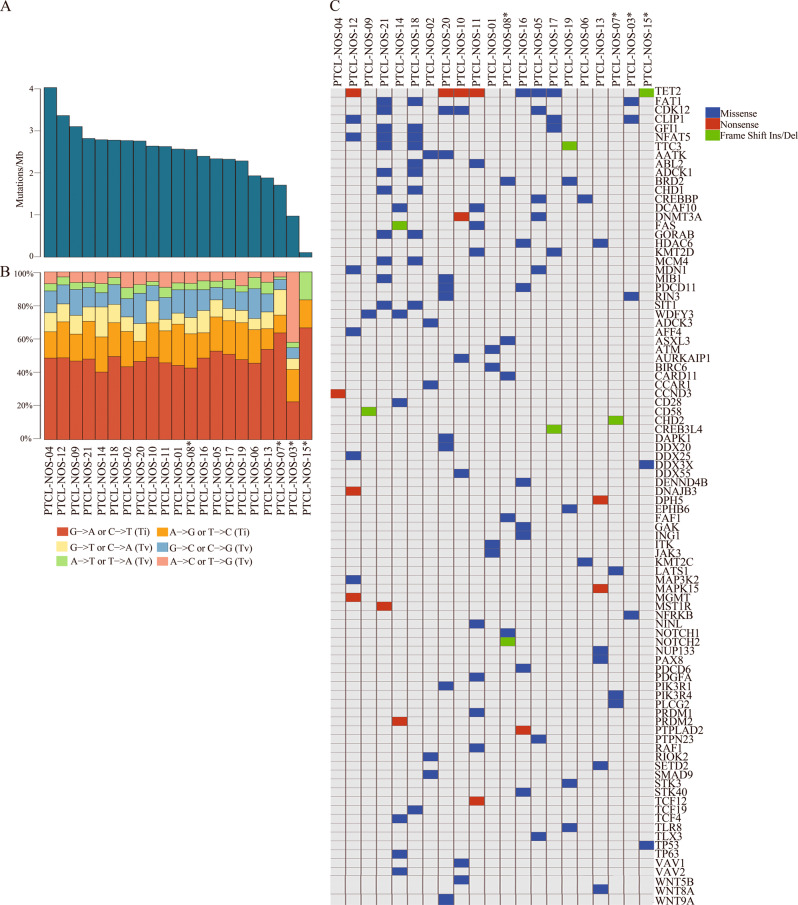
Mutation numbers and spectrum within the peripheral T-cell lymphomas not otherwise specified discovery sample set. **a** The rate of nonsynonymous mutations is showed as mutations per megabase, with individual peripheral T-cell lymphoma not otherwise specified samples ranked by total number of mutations. **b** The rate of base substitution observed in each individual, Ti = transition; Tv = transversion. **c** Heatmap representing the distribution of mutations in 92 genes mutated in the 21 peripheral T-cell lymphomas not otherwise specified of the discovery set. Each row represents a gene and each column represents the samples. Only one mutation per gene is shown if multiple mutations were found in a sample. Asterisks indicate paired samples

The median number of nonsynonymous mutations per Mb was 2.5 (range, 0.11–4) (Fig. 1a) with a median of 135 mutations (range, 6–204) per patient.

The pattern of nucleotide substitutions revealed a predominance of transitions over transversions (1.676:857, ratio of 1.9) and a preferential targeting of G and C nucleotides (affecting G/C compared with A/T nucleotides) like the somatic variation spectrum in other cancers (Fig. 1b).

Considering variants with total depth in tumor >10×, we found 2026 variants affecting 1799 genes. These were filtered according to the following criteria: (1) transcribed putative somatic mutations detected in both whole exome sequencing and RNA-sequencing; (2) genes previously reported in the literature to be potentially relevant for lymphoma biology, and (3) for unpaired samples only genes that were mutated also in paired samples and/or relevant to lymphomagenesis.

Based on these criteria, we identified nonsynonymous coding variants in several genes associated with chromatin structure modification and epigenetic regulation (*TET2, DNMT3A, KMT2C, KMT2D, SETD2, CREBBP*), tumor suppression (*FAT1, LATS1, STK3, TP53, TP63, ATM*), and NOTCH signaling (*NOTCH1, NOTCH2*) (Fig. 1c).

### Targeted sequencing of peripheral T-cell lymphoma not otherwise specified identifies recurrent mutations

The whole coding exons of 137 genes, of which 92 identified by whole exome sequencing and 45 chosen from literature (see material and methods and Supplementary Table S2), were analyzed for their mutation recurrence by targeted sequencing in both the extension and discovery sets.

Samples were sequenced, achieving an 800× coverage for more than 85% of the targeted regions for all the cases.

First, we validated selected mutations by MiSeq sequencing approach and obtained 89% validation rate (Supplementary Table S3).

Out of the 137 genes analyzed, the variants were selected if absent in normal saliva samples and were predicted to have functional consequences by means of at least four out of nine structure/homology-based tools (Polyphen2, Polyphen2_HVAR, Polyphen2_HDIV, LRT_pred, MutationTaster_pred, FATHMM_pred, RadialSVM_pred, LR_pred, Mutation Assessor). Based on these criteria, we found 52 genes mutated in at least 2/71 cases (Supplementary Table S4).

As expected, genes coding proteins involved in chromatin/epigenetic regulation were recurrently mutated [5–7]. The most frequently mutated gene was *KMT2C* (*MLL3*), not previously described in peripheral T-cell lymphoma not otherwise specified, presenting nonsynonymous somatic mutations in 23/71 samples (32%). This gene is a member of the myeloid/lymphoid or mixed-lineage leukemia family and encodes a nuclear histone methyltransferase protein that regulates gene transcription by modifying the chromatin structure mediating mono- and tri-methylation of histone H3 at lysine 4 [23]. The majority of *KMT2C* mutations were highly biased toward missense events rather than frameshift insertions/deletions or nonsense mutations, and mainly occurred within the PHD-like zinc-binding (252–331 position) and PHD-finger (1009–1057 position) domains. Additional histone methylation and chromatin remodeling genes were mutated: *TET*2 (16/71, 22%), *KMT2D (*16/71, 22%*)*, *CREBBP (*11/71, 16%), *KMT2A* (8/71, 11%), *SETD2* (7/71, 10%) and *CHD1, MBD4* (5/71, 7%, each), *DNMT3A*, *ASXL3* (6/71, 8%, each).

We also found mutations in *NOTCH1/NOTCH2* and *JAK/STAT* genes, previously reported to be mutated in peripheral T-cell lymphomas[24, 25]: *NOTCH1* (16/71, 22%), *NOTCH2* (14/71, 19%), *JAK3* (5/71, 7%), and *STAT6* (2/71, 3%).

### Novel recurrent genetic alterations in peripheral T-cell lymphoma not otherwise specified

**Fig. 2:**
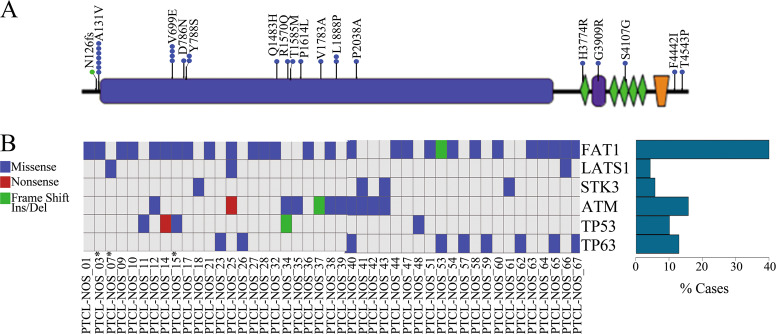
**a** Recurrent mutations in FAT1 gene. Graphical representation of FAT1 protein structure and the relative positions of mutations. **b** Heatmap representing the distribution of mutations in 6 tumor suppressor genes, each row represents a gene and each column represents the samples.Asterisks indicate paired samples

Targeted sequencing identified recurrent mutations of *FAT1* gene in 28/71 patients (39%). The gene encodes for the FAT atypical cadherin 1a type I transmembrane protein with 34 cadherin repeats, five epidermal growth factor-like repeats, and one laminin G motif on the extracellular side, followed by a transmembrane region and cytoplasmic domain. Most of the mutations were missense variants spanning across the gene in the cadherin repeats, epidermal growth factor-like, and laminin G-like domains (Fig. 2a). FAT1 acts as an adhesion molecule and/or signaling receptor, during developmental processes and cell communication [26]. *FAT1* has been reported recurrently mutated across multiple types of cancers, including T-cell acute lymphoblastic leukemia [27] and it appears to potently suppress cancer cell growth by binding beta-catenin and antagonizing the nuclear localization. Inactivation of FAT1 via mutation therefore promotes Wnt signaling and tumorigenesis and affects patient survival [28]. Moreover, FAT1 is also involved in the assembly and activation of the Hippo signalome leading to phosphorylation and inactivation of YAP1 [29].

Besides *FAT1* mutations, we found mutated also the following tumor suppressor genes: *LATS1* (3/71) and *STK3* (4/71) belonging to the core of hippo signaling, and *TP53* (5/71), *TP63* (9/71), *ATM* (11/71) (Fig. 2b). Since protein function depends on its structural stability, we performed a computational approach using INPS-MD to predict the impact of missense mutations on protein stability based on free energy change (ΔΔG) upon single point mutation. More than half of missense mutations were predicted to destabilize the FAT1 protein (ΔΔG < −0.5 Kcal/mol). Similarly, also two out of three missense mutations in *LATS1*, 3/4 in STK3, 2/3 in *TP53*, 3/5 in *TP63*, and 5/9 in *ATM*, appeared to affect protein stability with an ΔΔG < −0.5 Kcal/mol(Supplementary Table S5).

*FAT1* mutations spanned over the two main GATA3/TBX21 subgroups of peripheral T-cell lymphomas not otherwise specified (Supplementary Fig. S1 and Fig. S2, Supplementary Table S6). Moreover, no correlation was observed with any specific morphologic and/or phenotypic finding. Looking at the transcriptome, 100 genes were up and 109 genes downregulated (absolute FC > 2) between *FAT1* mutated (*N* = 8) versus *FAT1* wild-type cases (*N* = 10) (Supplementary Fig. S3, Supplementary Table S7).

*FAT1* mutated cases presented a signature enriched of genes involved in growth, apoptosis, cell migration, and invasiveness (FDR q-value ≤ 0.01) [30–32] (Supplementary Fig. S4)

### Potential clinical relevance of *FAT1* tumor suppressor gene mutations

Complete clinical information was available in 61/71 patients. The median follow-up of living patients was 12.5 months (range 1–78 months). The 3-year OS was 24%.

**Fig. 3:**
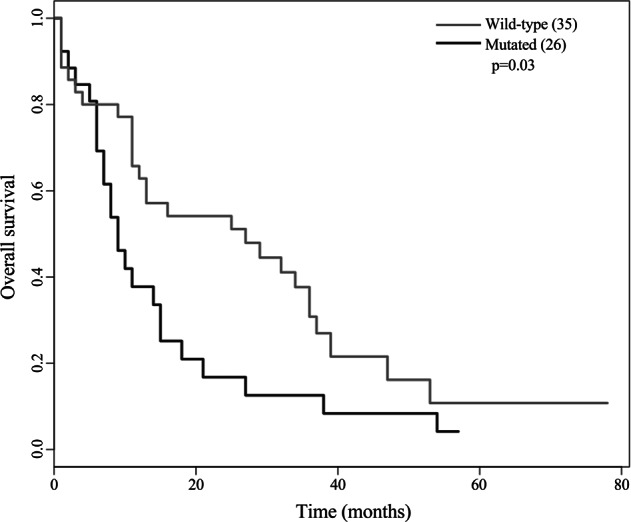
Survival analyses of *FAT1* mutated samples compared to *FAT1* wild type. The limit of significance for the log-rank Mantle–Cox test was defined as *P* < 0.05

Patients with mutations in *FAT1* tumor suppressor gene had a significantly reduced OS compared with individuals without mutations (median 11 months versus 26 months; *p* = 0.03, Fig. 3), while no effect on clinical outcome was observed for mutations in chromatin remodeling genes (*KMT2C, KMT2D, KMT2A, SETD2,* and *CHD1*) (Supplementary Fig. 5), in agreement with Meng-Meng Ji et al. [5].

These data suggest that mutations in *FAT1* tumor suppressor gene may represent a poor prognostic factor and might be involved in the pathogenesis of peripheral T-cell lymphoma not otherwise specified.

## Discussion

Peripheral T-cell lymphoma not otherwise specified is a heterogeneous group of nodal and extranodal mature T-cell lymphomas that do not correspond to any of the specifically defined T-cell entities in the revised 4th edition of the WHO classification [1].

To the best of our knowledge, the current study represents the largest whole exome sequencing investigation in peripheral T-cell lymphomas not otherwise specified. Based on the results of whole exome sequencing in the 21 cases of the discovery set and on data from the literature, a panel of 137 genes was constructed for targeted sequencing at high coverage, which was applied to the discovery set and 50 additional formalin-fixed paraffin embedded tissue samples.

We identified recurrent mutations in the epigenetic modifier genes *TET2, DNMT3A, KMT2C, CREBBP*, and *SETD2* with percentages like the ones reported in the literature [5–7]. In accordance with what observed in several other hematologic cancers, these mutations may represent early events occurring in the malignant transformation of hematopoietic stem cells. In addition, *ATM, TP53, TP63, NOTCH1, STAT3*, and *NOTCH2* mutations were identified. Conversely, no mutations were found in *RHOA, IDH2*, and *CD28*, known to occur in angioimmunoblastic T-cell lymphoma and other nodal T-cell lymphomas of T follicular helper origin.

In our comprehensive analysis, we found some unprecedented mutations such as the ones affecting *KMT2C* (*MLL3*) and *FAT1*. The latter, involving a tumor suppressor gene coding for a member of the cadherin superfamily, attracted our attention because of the high prevalence (39%). They were highly biased toward missense events rather than frameshift insertions/deletions or nonsense mutations, several of them being also reported in the COSMIC database [14]. As described in head and neck squamous cell carcinoma, FAT1 assembles a multimeric Hippo signaling complex resulting in the activation of core Hippo kinases [29]. We also observed genomic alterations in other members of the canonical Hippo pathway, including *LATS1*, and *SKT3/MTS-2*, although at a lower frequency than those affecting *FAT1*. These results suggest that the presence of somatic mutations in *FAT1* and Hippo core molecules might represent an important event at least in a percentage of peripheral T-cell lymphomas not otherwise specified, irrespective of the subgroup (GATA3 or TBX21-related) they belong to. Indeed, the majority of *FAT1* mutations were predicted to affect the protein stability, as demonstrated applying INPS-MD computational approach.

To understand the biological role of the observed *FAT1* alterations, we compared the transcriptome of cases with or without mutations. By supervised analysis, a specific gene signature of *FAT1* mutated cases was identified, which appeared significantly enriched in genes involved in cell growth and migration, apoptosis and invasiveness, all molecular pathways directly related to the gene function.

Importantly, *FAT1* mutations appeared to be clinically relevant in peripheral T-cell lymphomas not otherwise specified patients, being associated with poor prognosis.

In conclusion, the heterogeneity of peripheral T-cell lymphoma not otherwise specified remains a challenge to our understanding of disease pathobiology and clinical care of patients. In this work, using an integrated approach (whole exome sequencing, targeted sequencing and RNA-sequencing), we not only confirmed mutated genes previously described by others in peripheral T-cell lymphoma not otherwise specified but most importantly identified novel recurrent mutations in relevant genes, which seem to have implications on both functional and clinical grounds.

## Supplementary information

Supplementary_File
