# Correction: Whole exome sequencing reveals mutations in *FAT1* tumor suppressor gene clinically impacting on peripheral T-cell lymphoma not otherwise specified

**DOI:** 10.1038/s41379-019-0376-8

**Published:** 2019-09-26

**Authors:** Maria Antonella Laginestra, Luciano Cascione, Giovanna Motta, Fabio Fuligni, Claudio Agostinelli, Maura Rossi, Maria Rosaria Sapienza, Simona Righi, Alessandro Broccoli, Valentina Indio, Federica Melle, Valentina Tabanelli, Angelica Calleri, Domenico Novero, Fabio Facchetti, Giorgio Inghirami, Elena Sabattini, Francesco Bertoni, Stefano A. Pileri

**Affiliations:** 1 Department of Experimental, Diagnostic, and Specialty Medicine, University of Bologna, Bologna, Italy; 2 Università della Svizzera Italiana, Institute of Oncology Research, Bellinzona, Switzerland; 3 Division of Haematopathology, IEO European Institute of Oncology IRCCS, Milan, Italy; 4 Department of Genetics and Genome Biology, The Hospital for Sick Children, Toronto, ON Canada; 5 Division of Cancer Research Center “Giorgio Prodi” University of Bologna, Bologna, Italy; 6 Division of Pathological Anatomy, Quality and Safety of Diagnosis and Treatment, Città della Salute e della Scienza, Turin, Italy; 7 Division of Pathology Department of Molecular and Translational Medicine, Section of Pathology, University of Brescia, Brescia, Italy; 8 Department of Pathology and Laboratory Medicine, Weill Cornell Medical College, New York, NY USA

**Keywords:** T-cell lymphoma, Next-generation sequencing

**Correction to: Modern Pathology**

10.1038/s41379-019-0279-8

Since the publication of this paper, the authors noticed that the affiliation information for Francesco Bertolini, Giovanna Motta, Federica Melle, Valentina Tabanelli and Angelica Calleri was not complete. The correct affiliation is shown below:

Division of Haematopathology, IEO European Institute of Oncology IRCCS, Milan, Italy

Giovanna Motta, Federica Melle, Valentina Tabanelli, Angelica Calleri and Stefano A. Pileri.

