# Supplementary material for: Whole exome sequencing reveals mutations in FAT1 tumor suppressor gene clinically impacting on peripheral T-cell lymphoma not otherwise specified
Source: Mod Pathol. 2019 Apr 25;33(2):179–87. doi: 10.1038/s41379-019-0279-8 (PMC6994417; doi:10.1038/s41379-019-0279-8)

**Supplementary Information**

**Whole exome sequencing reveals mutations in *FAT1* tumor suppressor gene clinically impacting on Peripheral T-cell lymphoma not otherwise specified.**

Maria Antonella Laginestra^1*^, Luciano Cascione^2*^, Giovanna Motta^3,^ Fabio Fuligni^4^, Claudio Agostinelli^1^, Maura Rossi^1^, Maria Rosaria Sapienza^1^, Simona Righi^1^, Alessandro Broccoli^1^, Valentina Indio^5^, Federica Melle^3^, Valentina Tabanelli^3^, Angelica Calleri^3^, Domenico Novero^6^, Fabio Facchetti^7^, Giorgio Inghirami^8^, Elena Sabattini^1^, Francesco Bertoni^2+^ and Stefano A. Pileri^3+^.

* These authors contributed equally as first authors.

+ These authors contributed equally as last authors.

^1^ Department of Experimental, Diagnostic and Specialty Medicine, University of Bologna, Italy

^2^ Università della Svizzera italiana, Institute of Oncology Research, Bellinzona, Switzerland

^3^ Division of Haematopathology, European Institute of Oncology, Milan, Italy

^4^ Department of Genetics and Genome Biology, The Hospital for Sick Children, Toronto, Canada

^5^ Division of Cancer Research Center “Giorgio Prodi” University of Bologna, Bologna, Italy

^6^ Division of Pathological Anatomy, Quality and Safety of Diagnosis and Treatment, Città della Salute e della Scienza, Turin, Italy

^7^ Division of Pathology Department of Molecular and Translational Medicine,University of Brescia, Brescia

^8^ Department of Pathology and Laboratory Medicine, Weill Cornell Medical College, New York, NY, USA.

**Correspondence:** Maria Antonella Laginestra, Department of Experimental, Diagnostic and Specialty Medicine, University of Bologna, via Massarenti 9, Bologna, 40138, Italy; email: antolaginestra3@gmail.com and Stefano A. Pileri, Division of Haematopathology, European Institute of Oncology IRCCS, Via Ripamonti 435, Milano, 20121, Italy; email: stefano.pileri@ieo.it

**Supplementary Tables**

**Table S1:** Patients characteristics

| SAMPLE_ID | SEX | AGE | SITE | %TUMOR CELL | CD30 |
| --- | --- | --- | --- | --- | --- |
| PTCL/NOS_01 | M | 56 | LN | 95 | negative |
| PTCL/NOS_02 | F | 58 | LN | 75 | negative |
| PTCL/NOS_03* | M | 50 | LN | 75 | negative |
| PTCL/NOS_04 | F | 83 | LN | 80 | positive |
| PTCL/NOS_05 | M | 70 | LN | 75 | negative |
| PTCL/NOS_06 | F | 65 | LN | 75 | negative |
| PTCL/NOS_07* | M | 40 | LN | 95 | negative |
| PTCL/NOS_08* | M | 21 | LN | 75 | negative |
| PTCL/NOS_09 | F | 68 | LN | 80 | negative |
| PTCL/NOS_10 | F | 73 | LN | 75 | negative |
| PTCL/NOS_11 | F | 61 | LN | 75 | positive |
| PTCL/NOS_12 | M | 74 | LN | 75 | negative |
| PTCL/NOS_13 | F | 61 | LN | 75 | negative |
| PTCL/NOS_14 | F | 80 | LN | 75 | negative |
| PTCL/NOS_15* | M | 48 | LN | 75 | negative |
| PTCL/NOS_16 | F | 69 | LN | 75 | negative |
| PTCL/NOS_17 | M | 71 | LN | 75 | negative |
| PTCL/NOS_18 | F | 41 | LN | 75 | positive |
| PTCL/NOS_19 | F | 61 | LN | 75 | negative |
| PTCL/NOS_20 | M | 63 | LN | 75 | negative |
| PTCL/NOS_21 | M | 66 | LN | 95 | negative |
| PTCL/NOS_22 | F | 72 | LN | 75 | negative |
| PTCL/NOS_23 | M | 72 | LN | 80 | negative |
| PTCL/NOS_24 | F | 53 | LN | 85 | negative |
| PTCL/NOS_25 | M | 80 | LN | 75 | positive |
| PTCL/NOS_26 | M | 69 | LN | 90 | positive |
| PTCL/NOS_27 | F | 63 | LN | 90 | negative |
| PTCL/NOS_28 | M | 77 | LN | 90 | negative |
| PTCL/NOS_29 | F | 14 | LN | 90 | positive |
| PTCL/NOS_30 | F | 58 | LN | 75 | negative |
| PTCL/NOS_31 | M | 71 | LN | 90 | negative |
| PTCL/NOS_32 | M | 71 | LN | 75 | negative |
| PTCL/NOS_33 | M | 50 | LN | 90 | negative |
| PTCL/NOS_34 | M | 61 | LN | 90 | positive |
| PTCL/NOS_35 | M | 75 | LN | 90 | negative |
| PTCL/NOS_36 | F | 64 | LN | 95 | negative |
| PTCL/NOS_37 | F | 57 | LN | 85 | negative |
| PTCL/NOS_38 | M | 66 | LN | 90 | negative |
| PTCL/NOS_39 | M | 58 | LN | 90 | negative |
| PTCL/NOS_40 | M | 53 | LN | 75 | positive |
| PTCL/NOS_41 | F | 76 | LN | 80 | negative |
| PTCL/NOS_42 | M | 36 | LN | 90 | negative |
| PTCL/NOS_43 | M | 74 | LN | 75 | positive |
| PTCL/NOS_44 | M | 47 | LN | 80 | positive |
| PTCL/NOS_45 | F | 56 | LN | 75 | negative |
| PTCL/NOS_46 | M | 62 | LN | 90 | negative |
| PTCL/NOS_47 | F | 70 | LN | 75 | negative |
| PTCL/NOS_48 | M | 63 | LN | 75 | negative |
| PTCL/NOS_49 | F | 70 | LN | 90 | negative |
| PTCL/NOS_50 | M | 55 | LN | 80 | negative |
| PTCL/NOS_51 | M | 48 | LN | 80 | positive |
| PTCL/NOS_52 | F | 57 | LN | 90 | positive |
| PTCL/NOS_53 | F | 69 | LN | 90 | negative |
| PTCL/NOS_54 | M | 16 | LN | 75 | positive |
| PTCL/NOS_55 | M | 74 | LN | 80 | negative |
| PTCL/NOS_56 | F | 70 | LN | 85 | negative |
| PTCL/NOS_57 | M | 73 | LN | 75 | negative |
| PTCL/NOS_58 | M | 67 | LN | 90 | negative |
| PTCL/NOS_59 | M | 62 | LN | 75 | negative |
| PTCL/NOS_60 | M | 40 | LN | 80 | negative |
| PTCL/NOS_61 | M | 59 | LN | 85 | negative |
| PTCL/NOS_62 | M | 59 | LN | 95 | positive |
| PTCL/NOS_63 | M | 76 | LN | 90 | negative |
| PTCL/NOS_64 | F | 49 | LN | 80 | negative |
| PTCL/NOS_65 | M | 50 | LN | 75 | negative |
| PTCL/NOS_66 | F | 84 | LN | 95 | negative |
| PTCL/NOS_67 | M | 90 | LN | 90 | negative |
| PTCL/NOS_68 | M | 82 | LN | 80 | negative |
| PTCL/NOS_69 | F | 75 | LN | 75 | negative |
| PTCL/NOS_70 | F | 66 | LN | 95 | positive |
| PTCL/NOS_71 | F | 84 | LN | 90 | negative |

**Table S2:** 137 genes candidate for targeted sequencing

| **Genes identified by WES** | AATK, ABL2, ADCK1, ADCK3, AFF4, ASXL3, ATM, AURKAIP1, BIRC6, BRD2, CARD11, CCAR1, CCND3, CD28, CD58, CDK12, CHD1, CHD2, CLIP1, CREB3L4, CREBBP, DAPK1, DCAF10, DDX20, DDX25, DDX3X, DDX55, DENND4B, DNAJB3, DNMT3A, DPH5, EPHB6, FAF1, FAS, FAT1, GAK, GFI1, GORAB, HDAC6, ING1, ITK, JAK3, KMT2C, KMT2D, LATS1, MAP3K2, MAPK15, MCM4, MDN1, MGMT, MIB1, MST1R, NFAT5, NFRKB, NINL, NOTCH1, NOTCH2, NUP133, PAX8, PDCD11, PDCD6, PDGFA, PIK3R1, PIK3R4, PLCG2, PRDM1, PRDM2, PTPLAD2, PTPN23, RAF1, RIN3, RIOK2, SETD2, SIT1, SMAD9, STK3, STK40, TCF12, TCF19, TCF4, TET2, TLR8, TLX3, TP53, TP63, TTC3, VAV1, VAV2, WDFY3, WNT5B, WNT8A, WNT9A |
| --- | --- |
| **Genes reported mutated in PTCLs** | AKT1, B2M, BRAF, CALR, CCR4, CDK11B, CEP170, CTNNB1, DUSP22, ERBB2, FYN, GNB1, GTF2I, HSH2D, IDH1, IDH2, IRF4, JAK1, JAK2, KDM6A, KIT, KMT2A, KRAS, LCK, LRP6, MAPK3, MAPKAPK2, MBD4, MVP, MYC, MYD88, NFKBIA, NLRP4, PIK3CA, PIK3R5, PLCG1, PTPN7, PTPRC, RHOA, STAT3, STAT5B, STAT6, STX11, TNFRSF1B, TRAF6 |

**Table S3:** Validation of whole exome selected mutations by MiSeq

| **Sample_ID** | **Gene.ref** | **Chr** | **Genomic Coordinates** | **Wild-type allele** | **Mutated allele** | **aa_change** | **MiSeq Validation** |
| --- | --- | --- | --- | --- | --- | --- | --- |
| PTCL-NOS_02 | AATK | 17 | 79095055 | T | C | p.Q791R | not_validated |
| PTCL-NOS_20 | AATK | 17 | 79104971 | G | C | p.A75G | Validated |
| PTCL-NOS_11 | ABL2 | 1 | 179077404 | A | G | p.S979P | Validated |
| PTCL-NOS_18 | ABL2 | 1 | 179100578 | G | T | p.Q66K | Validated |
| PTCL-NOS_21 | ABL2 | 1 | 179100578 | G | T | p.Q66K | Validated |
| PTCL-NOS_21 | ADCK1 | 14 | 78392195 | A | G | p.Q298R | Validated |
| PTCL-NOS_18 | ADCK1 | 14 | 78392195 | A | G | p.Q298R | Validated |
| PTCL-NOS_02 | ADCK3 | 1 | 227174271 | G | A | p.V593I | Validated |
| PTCL-NOS_12 | AFF4 | 5 | 132232763 | C | A | p.G520V | Validated |
| PTCL-NOS_08* | ASXL3 | 18 | 31324034 | G | T | p.V1408F | Validated |
| PTCL-NOS_01 | ATM | 11 | 108214033 | G | A | p.D2785N | Validated |
| PTCL-NOS_10 | AURKAIP1 | 1 | 1309448 | G | A | p.R144W | Validated |
| PTCL-NOS_01 | BIRC6 | 2 | 32641069 | G | A | p.V904I | Validated |
| PTCL-NOS_08* | BRD2 | 6 | 32944574 | A | G | p.N354S | Validated |
| PTCL-NOS_19 | BRD2 | 6 | 32947909 | A | G | p.T669A | Validated |
| PTCL-NOS_08* | CARD11 | 7 | 2969689 | T | G | p.E530D | Validated |
| PTCL-NOS_02 | CCAR1 | 10 | 70525672 | A | G | p.I697V | Validated |
| PTCL-NOS_04 | CCND3 | 6 | 41903731 | G | A | p.Q204* | Validated |
| PTCL-NOS_14 | CD28 | 2 | 204591674 | A | T | p.D124V | Validated |
| PTCL-NOS_09 | CD58 | 1 | 117113535 | 0 | A | p.L20fs | Validated |
| PTCL-NOS_05 | CDK12 | 17 | 37686920 | C | T | p.P1266L | not_validated |
| PTCL-NOS_20 | CDK12 | 17 | 37686920 | C | T | p.P1266L | not_validated |
| PTCL-NOS_21 | CDK12 | 17 | 37686920 | C | T | p.P1266L | not_validated |
| PTCL-NOS_05 | CDK12 | 17 | 37618740 | A | G | p.K139R | Validated |
| PTCL-NOS_10 | CDK12 | 17 | 37686920 | C | T | p.P1266L | Validated |
| PTCL-NOS_18 | CDK12 | 17 | 37686920 | C | T | p.P1266L | Validated |
| PTCL-NOS_18 | CHD1 | 5 | 98236967 | T | G | p.R170S | Validated |
| PTCL-NOS_21 | CHD1 | 5 | 98236967 | T | G | p.R170S | Validated |
| PTCL-NOS_07* | CHD2 | 15 | 93518159 | C | 0 | p.H852fs | Validated |
| PTCL-NOS_03* | CLIP1 | 12 | 122825949 | T | C | p.E555G | Validated |
| PTCL-NOS_12 | CLIP1 | 12 | 122812630 | G | C | p.T992S | Validated |
| PTCL-NOS_17 | CLIP1 | 12 | 122825567 | C | A | p.M682I | Validated |
| PTCL-NOS_17 | CREB3L4 | 1 | 153941085 | G | 0 | p.L28fs | Validated |
| PTCL-NOS_05 | CREBBP | 16 | 3778424 | T | G | p.Q2170H | Validated |
| PTCL-NOS_06 | CREBBP | 16 | 3779115 | T | C | p.N1940S | Validated |
| PTCL-NOS_20 | DAPK1 | 9 | 90322088 | T | C | p.Y1368H | Validated |
| PTCL-NOS_14 | DCAF10 | 9 | 37861380 | C | T | p.R519W | Validated |
| PTCL-NOS_11 | DCAF10 | 9 | 37861380 | C | T | p.R519W | Validated |
| PTCL-NOS_20 | DDX20 | 1 | 112299307 | G | A | p.C114Y | Validated |
| PTCL-NOS_12 | DDX25 | 11 | 125775474 | G | A | p.D53N | Validated |
| PTCL-NOS_15* | DDX3X | X | 41206199 | C | T | p.P552L | Validated |
| PTCL-NOS_10 | DDX55 | 12 | 124086733 | C | T | p.P13L | Validated |
| PTCL-NOS_16 | DENND4B | 1 | 153912081 | G | T | p.N601K | not_validated |
| PTCL-NOS_12 | DNAJB3 | 2 | 234652385 | T | A | p.K60* | Validated |
| PTCL-NOS_05 | DNMT3A | 2 | 25464451 | G | A | p.R499C | Validated |
| PTCL-NOS_10 | DNMT3A | 2 | 25467032 | G | A | p.Q426* | Validated |
| PTCL-NOS_13 | DPH5 | 1 | 101458208 | G | A | p.R207* | Validated |
| PTCL-NOS_13 | DPH5 | 1 | 101456121 | G | A | p.A234V | Validated |
| PTCL-NOS_19 | EPHB6 | 7 | 142563286 | C | T | p.P43S | Validated |
| PTCL-NOS_08* | FAF1 | 1 | 51005347 | C | T | p.R441Q | Validated |
| PTCL-NOS_11 | FAS | 10 | 90770534 | T | G | p.L177R | Validated |
| PTCL-NOS_14 | FAS | 10 | 90768664 | ACTGC | 0 | p.118_119del | Validated |
| PTCL-NOS_03* | FAT1 | 4 | 187534283 | T | C | p.Q3148R | Validated |
| PTCL-NOS_18 | FAT1 | 4 | 187540040 | C | T | p.R2567H | Validated |
| PTCL-NOS_21 | FAT1 | 4 | 187540040 | C | T | p.R2567H | Validated |
| PTCL-NOS_16 | GAK | 4 | 870910 | G | A | p.R648W | Validated |
| PTCL-NOS_18 | GFI1 | 1 | 92946513 | G | C | p.A144G | not_validated |
| PTCL-NOS_21 | GFI1 | 1 | 92946513 | G | C | p.A144G | not_validated |
| PTCL-NOS_17 | GFI1 | 1 | 92941611 | C | T | p.R415Q | Validated |
| PTCL-NOS_18 | GORAB | 1 | 170521328 | G | A | p.D304N | Validated |
| PTCL-NOS_21 | GORAB | 1 | 170521328 | G | A | p.D304N | Validated |
| PTCL-NOS_13 | HDAC6 | X | 48681883 | C | T | p.S1025L | Validated |
| PTCL-NOS_16 | HDAC6 | X | 48674928 | A | G | p.H560R | Validated |
| PTCL-NOS_16 | ING1 | 13 | 111368118 | C | T | p.R110C | Validated |
| PTCL-NOS_01 | ITK | 5 | 156679654 | G | A | p.R610H | Validated |
| PTCL-NOS_01 | JAK3 | 19 | 17945696 | C | T | p.V722I | Validated |
| PTCL-NOS_06 | KMT2C | 7 | 151859683 | G | A | p.S3660L | Validated |
| PTCL-NOS_11 | KMT2D | 12 | 49448463 | C | T | p.R83Q | Validated |
| PTCL-NOS_17 | KMT2D | 12 | 49448463 | C | T | p.R83Q | Validated |
| PTCL-NOS_07* | LATS1 | 6 | 149997753 | C | T | p.C905Y | Validated |
| PTCL-NOS_12 | MAP3K2 | 2 | 128096623 | T | C | p.D3G | Validated |
| PTCL-NOS_13 | MAPK15 | 8 | 144803581 | G | T | p.E402* | Validated |
| PTCL-NOS_18 | MCM4 | 8 | 48882369 | G | A | p.A396T | Validated |
| PTCL-NOS_21 | MCM4 | 8 | 48882369 | G | A | p.A396T | Validated |
| PTCL-NOS_05 | MDN1 | 6 | 90450026 | T | A | p.E1507V | Validated |
| PTCL-NOS_12 | MDN1 | 6 | 90411291 | G | A | p.P2805S | Validated |
| PTCL-NOS_12 | MGMT | 10 | 131565253 | C | T | p.R237* | Validated |
| PTCL-NOS_18 | MIB1 | 18 | 19418345 | T | C | p.S617P | Validated |
| PTCL-NOS_20 | MIB1 | 18 | 19371415 | C | T | p.T330I | Validated |
| PTCL-NOS_21 | MIB1 | 18 | 19418345 | T | C | p.S617P | Validated |
| PTCL-NOS_18 | MST1R | 3 | 49934777 | G | A | p.Q707* | Validated |
| PTCL-NOS_21 | MST1R | 3 | 49934777 | G | A | p.Q707* | Validated |
| PTCL-NOS_12 | NFAT5 | 16 | 69711145 | C | T | p.H515Y | Validated |
| PTCL-NOS_18 | NFAT5 | 16 | 69726801 | A | T | p.T1007S | Validated |
| PTCL-NOS_21 | NFAT5 | 16 | 69726801 | A | T | p.T1007S | Validated |
| PTCL-NOS_03* | NFRKB | 11 | 129751661 | C | T | p.V452I | Validated |
| PTCL-NOS_11 | NINL | 20 | 25436325 | T | G | p.D1314A | not_validated |
| PTCL-NOS_08* | NOTCH1 | 9 | 139391805 | A | C | p.L2129R | Validated |
| PTCL-NOS_08* | NOTCH2 | 1 | 120612003 | GG | 0 | p.6_6del | Validated |
| PTCL-NOS_13 | NUP133 | 1 | 229600371 | G | A | p.L851F | Validated |
| PTCL-NOS_13 | PAX8 | 2 | 113994259 | C | T | p.D273N | Validated |
| PTCL-NOS_16 | PDCD11 | 10 | 105201601 | G | T | p.A1526S | Validated |
| PTCL-NOS_20 | PDCD11 | 10 | 105166474 | T | C | p.I266T | Validated |
| PTCL-NOS_16 | PDCD6 | 5 | 306746 | G | A | p.V80M | Validated |
| PTCL-NOS_11 | PDGFA | 7 | 552066 | G | C | p.L63V | Validated |
| PTCL-NOS_20 | PIK3R1 | 5 | 67522573 | T | G | p.L24V | Validated |
| PTCL-NOS_07* | PIK3R4 | 3 | 130405223 | G | A | p.H1103Y | Validated |
| PTCL-NOS_07* | PLCG2 | 16 | 81990412 | G | A | p.R1228Q | Validated |
| PTCL-NOS_11 | PRDM1 | 6 | 106554993 | G | T | p.V570F | Validated |
| PTCL-NOS_14 | PRDM2 | 1 | 14105142 | T | 0 | p.D83fs | Validated |
| PTCL-NOS_14 | PRDM2 | 1 | 14105139 | TG | 0 | p.82_83del | Validated |
| PTCL-NOS_16 | PTPLAD2 | 9 | 21026681 | G | A | p.R62* | not_validated |
| PTCL-NOS_05 | PTPN23 | 3 | 47452035 | A | G | p.Q916R | Validated |
| PTCL-NOS_11 | RAF1 | 3 | 12629100 | C | G | p.M469I | not_validated |
| PTCL-NOS_03* | RIN3 | 14 | 93118756 | A | T | p.K454N | Validated |
| PTCL-NOS_20 | RIN3 | 14 | 93142933 | G | C | p.A817P | Validated |
| PTCL-NOS_02 | RIOK2 | 5 | 96500763 | T | C | p.I496V | not_validated |
| PTCL-NOS_13 | SETD2 | 3 | 47162886 | C | T | p.M1080I | Validated |
| PTCL-NOS_18 | SIT1 | 9 | 35650215 | T | C | p.D108G | not_validated |
| PTCL-NOS_21 | SIT1 | 9 | 35650215 | T | C | p.D108G | not_validated |
| PTCL-NOS_02 | SMAD9 | 13 | 37439740 | G | A | p.L276F | Validated |
| PTCL-NOS_19 | STK3 | 8 | 99539049 | T | A | p.N302I | Validated |
| PTCL-NOS_16 | STK40 | 1 | 36807452 | G | C | p.F409L | Validated |
| PTCL-NOS_11 | TCF12 | 15 | 57565448 | C | T | p.Q462* | Validated |
| PTCL-NOS_18 | TCF19 | 6 | 31130399 | G | A | p.V315I | Validated |
| PTCL-NOS_21 | TCF19 | 6 | 31130399 | G | A | p.V315I | Validated |
| PTCL-NOS_14 | TCF4 | 18 | 52921814 | T | C | p.I262V | Validated |
| PTCL-NOS_11 | TET2 | 4 | 106155185 | C | G | p.P29R | Validated |
| PTCL-NOS_05 | TET2 | 4 | 106155185 | C | G | p.P29R | Validated |
| PTCL-NOS_10 | TET2 | 4 | 106158184 | G | T | p.E1029* | Validated |
| PTCL-NOS_11 | TET2 | 4 | 106155987 | T | A | p.C296* | Validated |
| PTCL-NOS_12 | TET2 | 4 | 106155185 | C | G | p.P29R | Validated |
| PTCL-NOS_12 | TET2 | 4 | 106156876 | C | T | p.Q593* | Validated |
| PTCL-NOS_15* | TET2 | 4 | 106197015 | A | 0 | p.Q1783fs | Validated |
| PTCL-NOS_16 | TET2 | 4 | 106155185 | C | G | p.P29R | Validated |
| PTCL-NOS_17 | TET2 | 4 | 106155185 | C | G | p.P29R | Validated |
| PTCL-NOS_20 | TET2 | 4 | 106156540 | C | T | p.Q481* | Validated |
| PTCL-NOS_19 | TLR8 | X | 12938553 | C | T | p.S465L | not_validated |
| PTCL-NOS_05 | TLX3 | 5 | 170737156 | G | A | p.A142T | not_validated |
| PTCL-NOS_15* | TP53 | 17 | 7577099 | C | T | p.R148K | Validated |
| PTCL-NOS_14 | TP63 | 3 | 189604298 | G | T | p.A395S | Validated |
| PTCL-NOS_19 | TTC3 | 21 | 38559416 | AG | 0 | p.1631_1632del | not_validated |
| PTCL-NOS_19 | TTC3 | 21 | 38494132 | T | C | p.C306R | Validated |
| PTCL-NOS_21 | TTC3 | 21 | 38494132 | T | C | p.C306R | Validated |
| PTCL-NOS_10 | VAV1 | 19 | 6854018 | G | C | p.R776P | Validated |
| PTCL-NOS_14 | VAV2 | 9 | 136633699 | G | C | p.I779M | Validated |
| PTCL-NOS_09 | WDFY3 | 4 | 85658516 | C | T | p.R2193H | Validated |
| PTCL-NOS_14 | WDFY3 | 4 | 85762326 | A | C | p.L132W | Validated |
| PTCL-NOS_10 | WNT5B | 12 | 1754981 | G | A | p.V215I | Validated |
| PTCL-NOS_13 | WNT8A | 5 | 137426719 | G | A | p.R338H | Validated |
| PTCL-NOS_20 | WNT9A | 1 | 228109409 | C | T | p.R303H | Validated |

**Table S4:** Genes identified by deep targeted sequencing

| **Sample_ID** | **Gene.ref** | **cDNA_Position** | **aa_change** | **Mutation_Type** |
| --- | --- | --- | --- | --- |
| PTCL/NOS_22 | ABL2 | c.G1445A | p.R482Q | missense |
| PTCL/NOS_23 | ABL2 | c.A1208G | p.K403R | missense |
| PTCL/NOS_24 | ABL2 | c.A1208G | p.K403R | missense |
| PTCL/NOS_30 | ADCK1 | c.663delC | p.R221fs | frameshift_del |
| PTCL/NOS_31 | ADCK1 | c.T353A | p.L118Q | missense |
| PTCL/NOS_23 | AFF4 | c.A2338G | p.K780E | missense |
| PTCL/NOS_25 | AFF4 | c.C1990A | p.Q664K | missense |
| PTCL/NOS_26 | AFF4 | c.C1990A | p.Q664K | missense |
| PTCL/NOS_26 | AFF4 | c.A650G | p.D217G | missense |
| PTCL/NOS_26 | AFF4 | c.C1990A | p.Q664K | missense |
| PTCL/NOS_28 | AFF4 | c.C1990A | p.Q664K | missense |
| PTCL/NOS_29 | AFF4 | c.C1990A | p.Q664K | missense |
| PTCL/NOS_12 | ASXL3 | c.T6200G | p.L2067R | missense |
| PTCL/NOS_32 | ASXL3 | c.A91G | p.K31E | missense |
| PTCL/NOS_33 | ASXL3 | c.A5366T | p.K1789I | missense |
| PTCL/NOS_34 | ASXL3 | c.C2213T | p.S738F | missense |
| PTCL/NOS_35 | ASXL3 | c.C4308A | p.S1436R | missense |
| PTCL/NOS_36 | ASXL3 | c.A6518G | p.K2173R | missense |
| PTCL/NOS_12 | ATM | c.C146G | p.S49C | missense |
| PTCL/NOS_25 | ATM | c.C4820T | p.P1607L | stopgain |
| PTCL/NOS_34 | ATM | c.T1448C | p.L483P | missense |
| PTCL/NOS_35 | ATM | c.A6637G | p.K2213E | missense |
| PTCL/NOS_37 | ATM | c.3984delG | p.L1328fs | frameshift_del |
| PTCL/NOS_38 | ATM | c.C2686T | p.L896F | missense |
| PTCL/NOS_39 | ATM | c.T3881C | p.I1294T | missense |
| PTCL/NOS_40 | ATM | c.T5867A | p.L1956H | missense |
| PTCL/NOS_41 | ATM | c.T5867A | p.L1956H | missense |
| PTCL/NOS_42 | ATM | c.A7738G | p.R2580G | missense |
| PTCL/NOS_43 | ATM | c.G8561A | p.R2854H | missense |
| PTCL/NOS_19 | BIRC6 | c.C5207T | p.P1736L | missense |
| PTCL/NOS_32 | BIRC6 | c.T13978C | p.Y4660H | missense |
| PTCL/NOS_44 | BIRC6 | c.C1592T | p.T531I | missense |
| PTCL/NOS_45 | BIRC6 | c.A9314G | p.Q3105R | missense |
| PTCL/NOS_46 | BIRC6 | c.T11291C | p.V3764A | missense |
| PTCL/NOS_37 | BRAF | c.G2177A | p.R726H | missense |
| PTCL/NOS_47 | BRAF | c.G2275A | p.G759R | missense |
| PTCL/NOS_25 | CCR4 | c.T761C | p.F254S | missense |
| PTCL/NOS_48 | CCR4 | c.G359A | p.G120D | missense |
| PTCL/NOS_33 | CDK12 | c.C1760T | p.S587F | missense |
| PTCL/NOS_41 | CDK12 | c.C185T | p.S62F | missense |
| PTCL/NOS_50 | CDK12 | c.3519delG | p.M1173fs | frameshift_del |
| PTCL/NOS_27 | CHD1 | c.G5114A | p.W1705* | stopgain |
| PTCL/NOS_28 | CHD1 | c.C2777G | p.A926G | missense |
| PTCL/NOS_18 | CHD1 | c.T88C | p.S30P | missense |
| PTCL/NOS_44 | CHD1 | c.T890A | p.V297D | missense |
| PTCL/NOS_29 | CHD2 | c.G3814T | p.G1272W | missense |
| PTCL/NOS_52 | CHD2 | c.C1183T | p.Q395* | missense |
| PTCL/NOS_36 | CHD3 | c.C592T | p.L198F | missense |
| PTCL/NOS_53 | CHD3 | c.C592T | p.L198F | missense |
| PTCL/NOS_37 | CREBBP | c.2543dupC | p.T848fs | frameshift_ins |
| PTCL/NOS_05 | CREBBP | c.C2771T | p.S924F | missense |
| PTCL/NOS_43 | CREBBP | c.C1435T | p.Q479* | stopgain |
| PTCL/NOS_44 | CREBBP | c.A2198C | p.Q733P | missense |
| PTCL/NOS_45 | CREBBP | c.2543dupC | p.T848fs | frameshift_ins |
| PTCL/NOS_46 | CREBBP | c.T5435C | p.L1812P | missense |
| PTCL/NOS_47 | CREBBP | c.C2771T | p.S924F | missense |
| PTCL/NOS_53 | CREBBP | c.C2771T | p.S924F | missense |
| PTCL/NOS_54 | CREBBP | c.G4996A | p.V1666I | missense |
| PTCL/NOS_55 | CREBBP | c.C5430A | p.H1810Q | missense |
| PTCL/NOS_56 | CREBBP | c.C1435T | p.Q479* | stopgain |
| PTCL/NOS_34 | DAPK1 | c.C187T | p.R63W | missense |
| PTCL/NOS_39 | DAPK1 | c.A3470T | p.H1157L | missense |
| PTCL/NOS_58 | DAPK1 | c.T4291G | p.*1431G | stoploss |
| PTCL/NOS_59 | DAPK1 | c.A3790T | p.K1264* | missense |
| PTCL/NOS_60 | DAPK1 | c.C4156T | p.R1386W | missense |
| PTCL/NOS_61 | DAPK1 | c.C4163T | p.A1388V | stopgain |
| PTCL/NOS_03* | DDX20 | c.C1736G | p.S579C | missense |
| PTCL/NOS_20 | DDX20 | c.G341A | p.C114Tyr | missense |
| PTCL/NOS_23 | DDX20 | c.T2032C | p.S678P | missense |
| PTCL/NOS_03* | DDX55 | c.G301C | p.V101L | missense |
| PTCL/NOS_06 | DDX55 | c.G301C | p.V101L | missense |
| PTCL/NOS_17 | DDX55 | c.G301C | p.V101L | missense |
| PTCL/NOS_62 | DDX55 | c.G275A | p.R92Q | missense |
| PTCL/NOS_10 | DNMT3A | c.T1766A | p.V589E | missense |
| PTCL/NOS_32 | DNMT3A | c.G326A | p.G109E | missense |
| PTCL/NOS_39 | DNMT3A | c.C662A | p.A221D | missense |
| PTCL/NOS_40 | DNMT3A | c.A2003G | p.D668G | missense |
| PTCL/NOS_45 | DNMT3A | c.A275T | p.E92V | missense |
| PTCL/NOS_54 | DNMT3A | c.A2003G | p.D668G | missense |
| PTCL/NOS_33 | DPH5 | c.209delA | p.K70fs | frameshift_del |
| PTCL/NOS_56 | DPH5 | c.T446A | p.V149E | missense |
| PTCL/NOS_38 | DUSP22 | c.A157C | p.I53L | missense |
| PTCL/NOS_41 | DUSP22 | c.160_161insA | p.P54fs | frameshift_ins |
| PTCL/NOS_41 | DUSP22 | c.A157C | p.I53L | missense |
| PTCL/NOS_19 | EPHB6 | c.C1003T | p.P335S | missense |
| PTCL/NOS_25 | EPHB6 | c.G1063A | p.D355N | missense |
| PTCL/NOS_29 | EPHB6 | c.T1898A | p.L633Q | missense |
| PTCL/NOS_55 | EPHB6 | c.A689T | p.Y230F | missense |
| PTCL/NOS_61 | EPHB6 | c.840delC | p.S280fs | frameshift_del |
| PTCL/NOS_01 | FAT1 | c.C392T | p.A131V | missense |
| PTCL/NOS_03* | FAT1 | c.C4754T | p.T1585M | missense |
| PTCL-NOS_03* | FAT1 | c.A9443G | p.Q3148R | missense |
| PTCL/NOS_09 | FAT1 | c.C392T | p.A131V | missense |
| PTCL/NOS_10 | FAT1 | c.C392T | p.A131V | missense |
| PTCL/NOS_12 | FAT1 | c.C392T | p.A131V | missense |
| PTCL/NOS_14 | FAT1 | c.C392T | p.A131V | missense |
| PTCL/NOS_15* | FAT1 | c.C392T | p.A131V | missense |
| PTCL/NOS_17 | FAT1 | c.C392T | p.A131V | missense |
| PTCL/NOS_17 | FAT1 | c.C4841T | p.P1614L | missense |
| PTCL/NOS_21 | FAT1 | c.C392T | p.A131V | missense |
| PTCL/NOS_25 | FAT1 | c.T2096A | p.V699E | missense |
| PTCL/NOS_27 | FAT1 | c.G4709A | p.R1570Q | missense |
| PTCL/NOS_28 | FAT1 | c.T13324A | p.F4442I | missense |
| PTCL/NOS_32 | FAT1 | c.T5663C | p.L1888P | missense |
| PTCL/NOS_36 | FAT1 | c.G4449T | p.Q1483H | missense |
| PTCL/NOS_38 | FAT1 | c.T5663C | p.L1888P | missense |
| PTCL/NOS_40 | FAT1 | c.T5348C | p.V1783A | missense |
| PTCL/NOS_44 | FAT1 | c.G2356A | p.D786N | missense |
| PTCL/NOS_47 | FAT1 | c.A2363C | p.Y788S | missense |
| PTCL/NOS_51 | FAT1 | c.T2096A | p.V699E | missense |
| PTCL/NOS_53 | FAT1 | c.377delA | p.N126fs | frameshift_del |
| PTCL/NOS_54 | FAT1 | c.A13627C | p.T4543P | missense |
| PTCL/NOS_58 | FAT1 | c.A2363C | p.Y788S | missense |
| PTCL/NOS_60 | FAT1 | c.A11321G | p.H3774R | missense |
| PTCL/NOS_63 | FAT1 | c.C6112G | p.P2038A | missense |
| PTCL/NOS_64 | FAT1 | c.G11725A | p.G3909R | missense |
| PTCL/NOS_65 | FAT1 | c.A12319G | p.S4107G | missense |
| PTCL/NOS_66 | FAT1 | c.T2096A | p.V699E | missense |
| PTCL/NOS_67 | FAT1 | c.T2096A | p.V699E | missense |
| PTCL/NOS_15* | HDAC6 | c.G2495A | p.R832H | missense |
| PTCL/NOS_32 | HDAC6 | c.A1871T | p.N624I | missense |
| PTCL/NOS_64 | HDAC6 | c.T1778A | p.V593E | missense |
| PTCL/NOS_24 | IDH1 | c.G54A | p.M18I | missense |
| PTCL/NOS_44 | IDH1 | c.G290A | p.G97D | missense |
| PTCL/NOS_08* | ING1 | c.C233G | p.S78C | missense |
| PTCL/NOS_27 | ING1 | c.G574A | p.E192K | missense |
| PTCL/NOS_55 | ING1 | c.G574A | p.E192K | missense |
| PTCL/NOS_63 | ING1 | c.G574A | p.E192K | missense |
| PTCL/NOS_64 | ING1 | c.C260T | p.S87F | missense |
| PTCL/NOS_67 | ING1 | c.G574A | p.E192K | missense |
| PTCL/NOS_30 | JAK3 | c.T2621A | p.I874N | missense |
| PTCL/NOS_31 | JAK3 | c.G2854C | p.A952P | missense |
| PTCL/NOS_36 | JAK3 | c.C1969T | p.R657W | missense |
| PTCL/NOS_66 | JAK3 | c.2230delC | p.L744fs | frameshift_del |
| PTCL/NOS_68 | JAK3 | c.2230delC | p.L744fs | frameshift_del |
| PTCL/NOS_23 | KMT2A | c.A3497C | p.D1166A | missense |
| PTCL/NOS_28 | KMT2A | c.G6988A | p.G2330R | missense |
| PTCL/NOS_30 | KMT2A | c.C6902T | p.S2301F | missense |
| PTCL/NOS_47 | KMT2A | c.7781_7782insG | p.L2594fs | frameshift_ins |
| PTCL/NOS_52 | KMT2A | c.G9037A | p.G3013S | missense |
| PTCL/NOS_56 | KMT2A | c.G4531A | p.G1511R | missense |
| PTCL/NOS_57 | KMT2A | c.G4531A | p.G1511R | missense |
| PTCL/NOS_69 | KMT2A | c.G5057C | p.R1686P | missense |
| PTCL/NOS_01 | KMT2C | c.G2512A | p.G838S | missense |
| PTCL/NOS_01 | KMT2C | c.A2917G | p.R973G | missense |
| PTCL/NOS_01 | KMT2C | c.G943A | p.G315S | missense |
| PTCL/NOS_01 | KMT2C | c.C871T | p.L291F | missense |
| PTCL/NOS_01 | KMT2C | c.G2963T | p.C988F | missense |
| PTCL/NOS_02 | KMT2C | c.A2917G | p.R973G | missense |
| PTCL/NOS_02 | KMT2C | c.G2512A | p.G838S | missense |
| PTCL/NOS_02 | KMT2C | c.C925T | p.P309S | missense |
| PTCL/NOS_02 | KMT2C | c.C871T | p.L291F | missense |
| PTCL/NOS_02 | KMT2C | c.G943T | p.315Cy | missense |
| PTCL/NOS_02 | KMT2C | c.T2959C | p.Y987H | missense |
| PTCL/NOS_02 | KMT2C | c.G2963T | p.C988F | missense |
| PTCL/NOS_03* | KMT2C | c.G2512A | p.G838S | missense |
| PTCL/NOS_03* | KMT2C | c.A2917G | p.R973G | missense |
| PTCL/NOS_03* | KMT2C | c.C925T | p.P309S | missense |
| PTCL/NOS_03* | KMT2C | c.G943A | p.G315S | missense |
| PTCL/NOS_03* | KMT2C | c.C871T | p.L291F | missense |
| PTCL/NOS_03* | KMT2C | c.G2963T | p.C988F | missense |
| PTCL/NOS_06 | KMT2C | c.G2512A | p.G838S | missense |
| PTCL/NOS_06 | KMT2C | c.A2917G | p.R973G | missense |
| PTCL/NOS_06 | KMT2C | c.C871T | p.L291F | missense |
| PTCL/NOS_06 | KMT2C | c.T2959C | p.Y987H | missense |
| PTCL/NOS_06 | KMT2C | c.G2963T | p.C988F | missense |
| PTCL/NOS_07* | KMT2C | c.A2917G | p.F973G | missense |
| PTCL/NOS_07* | KMT2C | c.G2512A | p.G838 | missense |
| PTCL/NOS_07* | KMT2C | c.C871T | p.L291F | missense |
| PTCL/NOS_07* | KMT2C | c.T2959C | p.Y987H | missense |
| PTCL/NOS_07* | KMT2C | c.G2963T | p.C988F | missense |
| PTCL/NOS_08* | KMT2C | c.A2917G | p.R973G | missense |
| PTCL/NOS_08* | KMT2C | c.G2512A | p.G838S | missense |
| PTCL/NOS_08* | KMT2C | c.C925T | p.P309S | missense |
| PTCL/NOS_08* | KMT2C | c.C871T | p.L291F | missense |
| PTCL/NOS_08* | KMT2C | c.T2959C | p.Y987H | missense |
| PTCL/NOS_08* | KMT2C | c.G2963T | p.C988F | missense |
| PTCL/NOS_09 | KMT2C | c.G5053T | p.A1685S | missense |
| PTCL/NOS_09 | KMT2C | c.A2917G | p.R973G | missense |
| PTCL/NOS_09 | KMT2C | c.G2512A | p.G838S | missense |
| PTCL/NOS_09 | KMT2C | c.G943A | p.G315S | missense |
| PTCL/NOS_09 | KMT2C | c.C871T | p.L291F | missense |
| PTCL/NOS_09 | KMT2C | c.G2963T | p.C988F | missense |
| PTCL/NOS_10 | KMT2C | c.G2512A | p.G838S | missense |
| PTCL/NOS_10 | KMT2C | c.A2917G | p.R973G | missense |
| PTCL/NOS_10 | KMT2C | c.G943A | p.G315S | missense |
| PTCL/NOS_10 | KMT2C | c.C871T | p.L291F | missense |
| PTCL/NOS_10 | KMT2C | c.G2963T | p.C988F | missense |
| PTCL/NOS_11 | KMT2C | c.A2917G | p.R973G | missense |
| PTCL/NOS_11 | KMT2C | c.G2512A | p.G838S | missense |
| PTCL/NOS_11 | KMT2C | c.C925T | p.P309S | missense |
| PTCL/NOS_11 | KMT2C | c.G943A | p.G315S | missense |
| PTCL/NOS_11 | KMT2C | c.C871T | p.L291F | missense |
| PTCL/NOS_11 | KMT2C | c.G2963T | p.C988F | missense |
| PTCL/NOS_12 | KMT2C | c.A2917G | p.R973G | missense |
| PTCL/NOS_12 | KMT2C | c.G2512A | p.G838S | missense |
| PTCL/NOS_12 | KMT2C | c.C925T | p.P309S | missense |
| PTCL/NOS_12 | KMT2C | c.G943A | p.G315S | missense |
| PTCL/NOS_12 | KMT2C | c.C871T | p.L291F | missense |
| PTCL/NOS_12 | KMT2C | c.G2963T | p.C988F | missense |
| PTCL/NOS_13 | KMT2C | c.A2917G | p.R973G | missense |
| PTCL/NOS_13 | KMT2C | c.G2512A | p.G838S | missense |
| PTCL/NOS_13 | KMT2C | c.C871T | p.L291F | missense |
| PTCL/NOS_13 | KMT2C | c.G943A | p.G315C | missense |
| PTCL/NOS_13 | KMT2C | c.T2959C | p.Y987H | missense |
| PTCL/NOS_13 | KMT2C | c.G2963T | p.C988F | missense |
| PTCL/NOS_14 | KMT2C | c.G2512A | p.G838S | missense |
| PTCL/NOS_14 | KMT2C | c.C871T | p.L291F | missense |
| PTCL/NOS_14 | KMT2C | c.T2959C | p.Y987H | missense |
| PTCL/NOS_14 | KMT2C | c.G2963T | p.C988F | missense |
| PTCL/NOS_17 | KMT2C | c.A2917G | p.R973G | missense |
| PTCL/NOS_17 | KMT2C | c.G2512A | p.G838S | missense |
| PTCL/NOS_17 | KMT2C | c.G943A | p.G315S | missense |
| PTCL/NOS_17 | KMT2C | c.C871T | p.L291F | missense |
| PTCL/NOS_17 | KMT2C | c.G2963T | p.C988F | missense |
| PTCL/NOS_18 | KMT2C | c.C871T | p.L291F | missense |
| PTCL/NOS_18 | KMT2C | c.G2963T | p.C988F | missense |
| PTCL/NOS_19 | KMT2C | c.A2917G | p.R973G | missense |
| PTCL/NOS_19 | KMT2C | c.G2512A | p.G838S | missense |
| PTCL/NOS_19 | KMT2C | c.C871T | p.L291F | missense |
| PTCL/NOS_19 | KMT2C | c.T2959C | p.Y987H | missense |
| PTCL/NOS_19 | KMT2C | c.G2963T | p.C988F | missense |
| PTCL/NOS_21 | KMT2C | c.A2917G | p.R973G | missense |
| PTCL/NOS_21 | KMT2C | c.G2512A | p.G838S | missense |
| PTCL/NOS_21 | KMT2C | c.G943A | p.G315S | missense |
| PTCL/NOS_21 | KMT2C | c.C871T | p.L291F | missense |
| PTCL/NOS_21 | KMT2C | c.G2963T | p.C988F | missense |
| PTCL/NOS_22 | KMT2C | c.T10238A | p.I3413N | missense |
| PTCL/NOS_41 | KMT2C | c.A3696T | p.E1232D | missense |
| PTCL/NOS_46 | KMT2C | c.T14725G | p.W4909G | missense |
| PTCL/NOS_57 | KMT2C | c.G7498C | p.V2500L | missense |
| PTCL/NOS_59 | KMT2C | c.8390delA | p.K2797fs | missense |
| PTCL/NOS_61 | KMT2C | c.8916delT | p.N2972fs | frameshift_del |
| PTCL/NOS_68 | KMT2C | c.A1943G | p.E648G | missense |
| PTCL/NOS_11 | KMT2D | c.A5036C | p.E1679A | missense |
| PTCL/NOS_17 | KMT2D | c.T4979C | p.M1660T | missense |
| PTCL/NOS_25 | KMT2D | c.A5036C | p.E1679A | missense |
| PTCL/NOS_27 | KMT2D | c.A5036C | p.E1679A | missense |
| PTCL/NOS_31 | KMT2D | c.A5036C | p.E1679A | missense |
| PTCL/NOS_36 | KMT2D | c.16584_16585insT | p.A5529fs | frameshift_ins |
| PTCL/NOS_36 | KMT2D | c.2851dupC | p.L951fs | frameshift_ins |
| PTCL/NOS_36 | KMT2D | c.A9143G | p.D3048G | missense |
| PTCL/NOS_37 | KMT2D | c.3329_3330insT | p.P1110fs | frameshift_ins |
| PTCL/NOS_38 | KMT2D | c.A16334G | p.E5445G | missense |
| PTCL/NOS_38 | KMT2D | c.C16329A | p.Y5443* | stopgain |
| PTCL/NOS_40 | KMT2D | c.G13322A | p.G4441E | missense |
| PTCL/NOS_44 | KMT2D | c.C11050T | p.Q3684* | stopgain |
| PTCL/NOS_47 | KMT2D | c.G13322A | p.G4441E | missense |
| PTCL/NOS_47 | KMT2D | c.A5036C | p.E1679A | missense |
| PTCL/NOS_51 | KMT2D | c.C12862T | p.R4288W | missense |
| PTCL/NOS_54 | KMT2D | c.3329_3330insT | p.P1110fs | frameshift_ins |
| PTCL/NOS_54 | KMT2D | c.A5036C | p.E1679A | missense |
| PTCL/NOS_62 | KMT2D | c.G9085A | p.E3029K | missense |
| PTCL/NOS_62 | KMT2D | c.A5036C | p.E1679A | missense |
| PTCL/NOS_66 | KMT2D | c.2851dupC | p.L951fs | frameshift_ins |
| PTCL/NOS_66 | KMT2D | c.A5036C | p.E1679A | missense |
| PTCL/NOS_69 | KMT2D | c.A12071T | p.K4024M | missense |
| PTCL/NOS_07* | LATS1 | c.G2714A | p.C905Y | missense |
| PTCL/NOS_25 | LATS1 | c.T1625G | p.V542G | missense |
| PTCL/NOS_66 | LATS1 | c.G3376A | p.D1126N | missense |
| PTCL/NOS_37 | MAPK15 | c.855delG | p.L285fs | frameshift_del |
| PTCL/NOS_51 | MAPK15 | c.T269A | p.L90Q | missense |
| PTCL/NOS_53 | MAPK15 | c.T269A | p.L90Q | missense |
| PTCL/NOS_64 | MAPK15 | c.T269A | p.L90Q | missense |
| PTCL/NOS_11 | MBD4 | c.939delA | p.K313fs | missense |
| PTCL/NOS_32 | MBD4 | c.939delA | p.K313fs | frameshift_del |
| PTCL/NOS_52 | MBD4 | c.939delA | p.K313fs | frameshift_del |
| PTCL/NOS_56 | MBD4 | c.939delA | p.K313fs | frameshift_del |
| PTCL/NOS_56 | MBD4 | c.C539T | p.T180I | missense |
| PTCL/NOS_65 | MBD4 | c.939delA | p.K313fs | frameshift_del |
| PTCL/NOS_67 | MBD4 | c.939delA | p.K313fs | frameshift_del |
| PTCL/NOS_01 | MDN1 | c.G9010A | p.E3004K | missense |
| PTCL/NOS_07* | MDN1 | c.G6784A | p.G2262R | missense |
| PTCL/NOS_11 | MDN1 | c.G9010A | p.E3004K | missense |
| PTCL/NOS_15* | MDN1 | c.G9010A | p.E3004K | missense |
| PTCL/NOS_23 | MDN1 | c.A16024C | p.K5342Q | missense |
| PTCL/NOS_48 | MDN1 | c.A2591T | p.D864V | missense |
| PTCL/NOS_50 | MDN1 | c.G1951C | p.A651P | missense |
| PTCL/NOS_01 | MST1R | c.C223A | p.R75S | missense |
| PTCL/NOS_32 | MST1R | c.A3872G | p.E1291G | missense |
| PTCL/NOS_45 | MST1R | c.C956T | p.P319L | missense |
| PTCL/NOS_10 | MYC | c.A77G | p.N26S | missense |
| PTCL/NOS_13 | MYC | c.A77G | p.N26S | missense |
| PTCL/NOS_08* | NOTCH1 | c.T6386G | p.L2129R | missense |
| PTCL/NOS_24 | NOTCH1 | c.G506T | p.S169I | missense |
| PTCL/NOS_27 | NOTCH1 | c.G2296T | p.G766C | missense |
| PTCL/NOS_28 | NOTCH1 | c.C4160T | p.A1387V | missense |
| PTCL/NOS_33 | NOTCH1 | c.C5690A | p.T1897K | missense |
| PTCL/NOS_36 | NOTCH1 | c.7379_7383del | p.E2460fs | frameshift_del |
| PTCL/NOS_38 | NOTCH1 | c.G2296T | p.G766C | missense |
| PTCL/NOS_39 | NOTCH1 | c.T4765C | p.S1589P | missense |
| PTCL/NOS_46 | NOTCH1 | c.7478dupC | p.P2493fs | frameshift_ins |
| PTCL/NOS_53 | NOTCH1 | c.A631C | p.T211P | stopgain |
| PTCL/NOS_55 | NOTCH1 | c.7379_7383del | p.E2460fs | frameshift_del |
| PTCL/NOS_55 | NOTCH1 | c.C6274G | p.H2092D | missense |
| PTCL/NOS_57 | NOTCH1 | c.G2296T | p.G766C | missense |
| PTCL/NOS_64 | NOTCH1 | c.G2296T | p.G766C | missense |
| PTCL/NOS_66 | NOTCH1 | c.G2296T | p.G766C | missense |
| PTCL/NOS_67 | NOTCH1 | c.G5972A | p.R1991H | missense |
| PTCL/NOS_69 | NOTCH1 | c.G506T | p.S169I | stopgain |
| PTCL/NOS_24 | NOTCH2 | c.C1289T | p.A430V | stopgain |
| PTCL/NOS_28 | NOTCH2 | c.A1418C | p.D473A | missense |
| PTCL/NOS_30 | NOTCH2 | c.G689A | p.C230Y | missense |
| PTCL/NOS_33 | NOTCH2 | c.A1418C | p.D473A | missense |
| PTCL/NOS_34 | NOTCH2 | c.T1413A | p.C471* | stopgain |
| PTCL/NOS_46 | NOTCH2 | c.G689A | p.C230Y | missense |
| PTCL/NOS_47 | NOTCH2 | c.G689A | p.C230Y | missense |
| PTCL/NOS_53 | NOTCH2 | c.G6056T | p.R2019L | missense |
| PTCL/NOS_55 | NOTCH2 | c.G689A | p.C230Y | missense |
| PTCL/NOS_56 | NOTCH2 | c.G6056T | p.R2019L | missense |
| PTCL/NOS_59 | NOTCH2 | c.A1678T | p.T560S | missense |
| PTCL/NOS_64 | NOTCH2 | c.A1418C | p.D473A | missense |
| PTCL/NOS_66 | NOTCH2 | c.G1526A | p.C509Y | missense |
| PTCL/NOS_70 | NOTCH2 | c.C6925T | p.L2309F | missense |
| PTCL/NOS_01 | PDCD11 | c.A5612C | p.D1871A | missense |
| PTCL/NOS_02 | PDCD11 | c.A5612C | p.D1871A | missense |
| PTCL/NOS_03* | PDCD11 | c.A5612C | p.D1871A | missense |
| PTCL/NOS_04 | PDCD11 | c.G4829T | p.R1610L | missense |
| PTCL/NOS_15* | PDCD11 | c.A133G | p.K45E | missense |
| PTCL/NOS_16 | PDCD11 | c.A5612C | p.D1871A | missense |
| PTCL/NOS_16 | PDCD11 | c.A133G | p.K45E | missense |
| PTCL/NOS_20 | PDCD11 | c.G4829T | p.R1610L | missense |
| PTCL/NOS_35 | PDGFA | c.T371G | p.V124G | missense |
| PTCL/NOS_67 | PDGFA | c.A400C | p.N134H | missense |
| PTCL/NOS_20 | PIK3R1 | c.T70G | p.L24V | missense |
| PTCL/NOS_36 | PIK3R1 | c.43delA | p.K15fs | frameshift_del |
| PTCL/NOS_34 | PLCG2 | c.T3796C | p.*1266Q | stoploss |
| PTCL/NOS_67 | PLCG2 | c.1087delG | p.G363fs | frameshift_del |
| PTCL/NOS_69 | PLCG2 | c.2383_2384insA | p.L795fs | frameshift_ins |
| PTCL/NOS_60 | PRDM2 | c.3856delA | p.K1286fs | frameshift_del |
| PTCL/NOS_63 | PRDM2 | c.3856delA | p.K1286fs | frameshift_del |
| PTCL/NOS_67 | PRDM2 | c.3856delA | p.K1286fs | frameshift_del |
| PTCL/NOS_36 | PTPN23 | c.2183delC | p.A728fs | frameshift_del |
| PTCL/NOS_39 | PTPN23 | c.3834delG | p.L1278fs | frameshift_del |
| PTCL/NOS_47 | PTPN23 | c.T77A | p.V26E | missense |
| PTCL/NOS_05 | RIOK2 | c.C287G | p.S96C | missense |
| PTCL/NOS_13 | RIOK2 | c.C287G | p.S96C | missense |
| PTCL/NOS_19 | RIOK2 | c.C287G | p.S96C | missense |
| PTCL/NOS_35 | RIOK2 | c.C1534T | p.Q512* | stopgain |
| PTCL/NOS_13 | SETD2 | c.A7057G | p.T2353A | missense |
| PTCL/NOS_26 | SETD2 | c.2931dupA | p.E978fs | frameshift_ins |
| PTCL/NOS_32 | SETD2 | c.2931dupA | p.E978fs | frameshift_ins |
| PTCL/NOS_52 | SETD2 | c.T5348G | p.M1783R | missense |
| PTCL/NOS_57 | SETD2 | c.2931dupA | p.E978fs | frameshift_ins |
| PTCL/NOS_66 | SETD2 | c.7162delA | p.T2388fs | frameshift_del |
| PTCL/NOS_66 | SETD2 | c.2931dupA | p.E978fs | frameshift_ins |
| PTCL/NOS_69 | SETD2 | c.2931dupA | p.E978fs | frameshift_ins |
| PTCL/NOS_31 | STAT6 | c.277delC | p.L93* | stopgain |
| PTCL/NOS_34 | STAT6 | c.C479G | p.T160S | missense |
| PTCL/NOS_18 | STK3 | c.C553T | p.P185S | missense |
| PTCL/NOS_41 | STK3 | c.C527T | p.A176V | missense |
| PTCL/NOS_43 | STK3 | c.G646A | p.E216K | missense |
| PTCL/NOS_61 | STK3 | c.A431G | p.H144R | missense |
| PTCL/NOS_14 | TCF4 | c.T891G | p.N297K | missense |
| PTCL/NOS_26 | TCF4 | c.17delT | p.F6fs | frameshift_del |
| PTCL/NOS_28 | TCF4 | c.17delT | p.F6fs | frameshift_del |
| PTCL/NOS_36 | TCF4 | c.A478C | p.T160P | missense |
| PTCL/NOS_41 | TCF4 | c.T20C | p.I7T | missense |
| PTCL/NOS_45 | TCF4 | c.A478C | p.T160P | missense |
| PTCL/NOS_61 | TCF4 | c.A478C | p.T160P | missense |
| PTCL/NOS_02 | TET2 | c.G1064A | p.G355D | missense |
| PTCL/NOS_15* | TET2 | c.C86G | p.Pro29Fs | frameshift_del |
| PTCL/NOS_05 | TET2 | c.C86G | p.P29F | missense |
| PTCL/NOS_06 | TET2 | c.T5162G | p.L1721W | missense |
| PTCL/NOS_09 | TET2 | c.T5162G | p.L1721W | missense |
| PTCL/NOS_10 | TET2 | c.T5162G | p.L1721* | stopgain |
| PTCL/NOS_11 | TET2 | c.C86G | p.P29R | missense |
| PTCL/NOS_16 | TET2 | c.C86G | p.P29R | missense |
| PTCL/NOS_17 | TET2 | c.C86G | p.P29R | missense |
| PTCL/NOS_30 | TET2 | c.C4099T | p.P1367S | missense |
| PTCL/NOS_32 | TET2 | c.2533_2534del | p.K845fs | frameshift_del |
| PTCL/NOS_38 | TET2 | c.C1199T | p.P400L | missense |
| PTCL/NOS_56 | TET2 | c.G2645A | p.C882Y | missense |
| PTCL/NOS_59 | TET2 | c.5330_5331del | p.L1777fs | frameshift_del |
| PTCL/NOS_62 | TET2 | c.T2327C | p.F776S | missense |
| PTCL/NOS_65 | TET2 | c.5264delA | p.E1755fs | frameshift_del |
| PTCL/NOS_38 | TLR8 | c.A700G | p.I234V | missense |
| PTCL/NOS_41 | TLR8 | c.C695T | p.T232I | missense |
| PTCL/NOS_62 | TLR8 | c.A2434G | p.K812E | missense |
| PTCL/NOS_69 | TLR8 | c.T1952C | p.L651P | missense |
| PTCL/NOS_11 | TP53 | c.C817T | p.R273C | missense |
| PTCL/NOS_14 | TP53 | c.C830G | p.P720* | stopgain |
| PTCL/NOS_15* | TP53 | c.C215G | p.R280K | missense |
| PTCL/NOS_34 | TP53 | c.49delT | p.S17fs | frameshift_del |
| PTCL/NOS_48 | TP53 | c.A311G | p.Q104C | missense |
| PTCL/NOS_23 | TP63 | c.A263G | p.K88R | missense |
| PTCL/NOS_26 | TP63 | c.C1164A | p.N388K | missense |
| PTCL/NOS_40 | TP63 | c.C370T | p.P124S | missense |
| PTCL/NOS_53 | TP63 | c.C368T | p.T123I | missense |
| PTCL/NOS_57 | TP63 | c.C1164A | p.N388K | missense |
| PTCL/NOS_59 | TP63 | c.C1164A | p.N388K | missense |
| PTCL/NOS_62 | TP63 | c.C1019T | p.T340M | missense |
| PTCL/NOS_65 | TP63 | c.C1164A | p.N388K | missense |
| PTCL/NOS_67 | TP63 | c.C1164A | p.N388K | missense |
| PTCL/NOS_02 | TTC3 | c.G2771A | p.R924H | missense |
| PTCL/NOS_03* | TTC3 | c.G2771A | p.R924H | missense |
| PTCL/NOS_13 | TTC3 | c.G2771A | p.R924H | missense |
| PTCL/NOS_18 | TTC3 | c.T916C | p.C306R | missense |
| PTCL/NOS_39 | TTC3 | c.A1487G | p.D496G | missense |
| PTCL/NOS_39 | TTC3 | c.G3806T | p.G1269V | missense |
| PTCL/NOS_39 | TTC3 | c.A4400G | p.Q1467R | missense |
| PTCL/NOS_58 | TTC3 | c.G3806T | p.G1269V | missense |

**Table S5:** INPS-MD tool to predict the impact of missense point mutations on protein stability based on free energy change (ΔΔG). Destabilizing mutations (∆∆G<-0.5 Kcal/mol), stabilizing mutations (∆∆G>0.5 Kcal/mol), neutral mutations (-0.5<=∆∆G<=0.5 Kcal/mol).

| ***FAT1*** | **Mutations** | **∆∆G** |
| --- | --- | --- |
|  | A131V | -0.947661 |
|  | V699E | -0.561008 |
|  | D786N | -0.164172 |
|  | Y788S | -2.104 |
|  | Q1483H | -0.366287 |
|  | R1570Q | -0.579443 |
|  | T1585M | -0.443257 |
|  | P1614L | -1.07188 |
|  | V1783A | -1.28912 |
|  | L1888P | -3.12543 |
|  | P2038A | -0.333422 |
|  | H3774R | -0.592024 |
|  | G3909R | -0.262584 |
|  | S4107G | -0.319085 |
|  | F4442I | -1.67544 |
|  | T4543P | -0.771335 |
| ***LATS1*** | V542G | -2.92752 |
|  | D1126N | -0.611538 |
|  | C905Y | -3.12562 |
| **STK3** | P185S | -3.57281 |
|  | A176V | 2.35624 |
|  | E216K | -0.120365 |
|  | H144R | -1.23569 |
| ***TP53*** | Q104C | -0.447158 |
|  | R273C | 0.369409 |
|  | R280K | -1.4840 |
| ***TP63*** | K88R | 0.0417787 |
|  | Q123I | 0.151228 |
|  | I124S | -3.09661 |
|  | F340M | -1.14499 |
|  | T388K | -0.488409 |
| ***ATM*** | S49C | -0.665712 |
|  | L483P | -1.47325 |
|  | L896F | 0.148811 |
|  | I1294T | -2.06696 |
|  | P1607L | -0.618062 |
|  | L1956H | -0.28742 |
|  | K2213E | -0.51713 |
|  | R2580G | -1.18033 |
|  | R2854H | -1.04495 |

**Table S6:** GATA3 and TBX21/T-bet Immunohistochemistry for cases belonging to extension set

| **Sample_ID** | **TBX21** | **GATA3** | **FAT1** |
| --- | --- | --- | --- |
| PTCL/NOS_51 | Positive | Negative | mutated |
| PTCL/NOS_25 | Negative | Positive | mutated |
| PTCL/NOS_40 | Positive | Positive | mutated |
| PTCL/NOS_28 | Positive | Positive | mutated |
| PTCL/NOS_64 | Positive | Positive | mutated |
| PTCL/NOS_60 | Negative | Positive | mutated |
| PTCL/NOS_67 | Positive | Positive | mutated |
| PTCL/NOS_53 | Positive | Positive | mutated |
| PTCL/NOS_44 | Positive | Positive | mutated |
| PTCL/NOS_63 | Negative | Positive | mutated |
| PTCL/NOS_34 | Positive | Positive | not mutated |
| PTCL/NOS_30 | Positive | Negative | not mutated |
| PTCL/NOS_49 | Positive | Positive | not mutated |
| PTCL/NOS_68 | Positive | Positive | not mutated |
| PTCL/NOS_35 | Positive | Positive | not mutated |
| PTCL/NOS_42 | Positive | Positive | not mutated |
| PTCL/NOS_57 | Positive | Positive | not mutated |
| PTCL/NOS_37 | Positive | Negative | not mutated |
| PTCL/NOS_22 | Positive | Positive | not mutated |
| PTCL/NOS_46 | Positive | Positive | not mutated |
| PTCL/NOS_41 | Positive | Positive | not mutated |
| PTCL/NOS_59 | Positive | Positive | not mutated |
| PTCL/NOS_69 | Positive | Positive | not mutated |
| PTCL/NOS_26 | Positive | Positive | not mutated |

**Table S7:** Differentially expressed genes between *FAT1* mutated vs. *FAT1* wild-type cases

| **Genes Up-regulated in FAT1 mutated vs. FAT1 wild-type cases** | | |
| --- | --- | --- |
| ***Ensembl_ID*** | ***gene_name*** | ***log_2_FC*** |
| ENSG00000136231.12 | IGF2BP3 | 4,1667185283 |
| ENSG00000159217.8 | IGF2BP1 | 3,789431519 |
| ENSG00000174255.6 | ZNF80 | 3,7458000703 |
| ENSG00000106178.5 | CCL24 | 3,7150968071 |
| ENSG00000213809.7 | KLRK1 | 3,6223108577 |
| ENSG00000188211.7 | NCR3LG1 | 3,5882259156 |
| ENSG00000160201.10 | U2AF1 | 3,5421033436 |
| ENSG00000180535.3 | BHLHA15 | 3,4524533323 |
| ENSG00000154165.4 | GPR15 | 3,376168306 |
| ENSG00000198146.4 | ZNF770 | 3,3080039237 |
| ENSG00000080200.8 | CRYBG3 | 3,2822171934 |
| ENSG00000163518.9 | FCRL4 | 3,24080844 |
| ENSG00000180509.10 | KCNE1 | 3,0873096561 |
| ENSG00000170128.3 | GPR25 | 3,0700522453 |
| ENSG00000171951.4 | SCG2 | 2,9560750273 |
| ENSG00000106236.3 | NPTX2 | 2,8841598863 |
| ENSG00000119608.11 | PROX2 | 2,878888908 |
| ENSG00000240184.5 | PCDHGC3 | 2,8317834012 |
| ENSG00000181418.7 | DDN | 2,8060733393 |
| ENSG00000186431.17 | FCAR | 2,789770011 |
| ENSG00000090382.5 | LYZ | 2,770780418 |
| ENSG00000181433.8 | SAGE1 | 2,7696531347 |
| ENSG00000156564.8 | LRFN2 | 2,7259888317 |
| ENSG00000213077.5 | FAM106A | 2,7015237073 |
| ENSG00000169313.9 | P2RY12 | 2,6847999107 |
| ENSG00000239264.7 | TXNDC5 | 2,6741143768 |
| ENSG00000087510.6 | TFAP2C | 2,6478741072 |
| ENSG00000161960.13 | EIF4A1 | 2,623797683 |
| ENSG00000117281.14 | CD160 | 2,6212701401 |
| ENSG00000156869.11 | FRRS1 | 2,6210788168 |
| ENSG00000009724.15 | MASP2 | 2,6096659201 |
| ENSG00000142178.7 | SIK1 | 2,5987756796 |
| ENSG00000169862.17 | CTNND2 | 2,5936798058 |
| ENSG00000090061.16 | CCNK | 2,5926531189 |
| ENSG00000176986.13 | SEC24C | 2,582669322 |
| ENSG00000180574.3 | EIF2S3L | 2,5376482353 |
| ENSG00000262406.2 | MMP12 | 2,5255324604 |
| ENSG00000109265.11 | KIAA1211 | 2,5249776539 |
| ENSG00000111732.9 | AICDA | 2,5130703275 |
| ENSG00000178502.5 | KLHL11 | 2,5111168312 |
| ENSG00000170927.13 | PKHD1 | 2,5012072188 |
| ENSG00000078098.12 | FAP | 2,4931023634 |
| ENSG00000147697.7 | GSDMC | 2,4923562171 |
| ENSG00000205730.6 | ITPRIPL2 | 2,4874291778 |
| ENSG00000076685.17 | NT5C2 | 2,4636593951 |
| ENSG00000198829.6 | SUCNR1 | 2,4608514892 |
| ENSG00000104970.9 | KIR3DX1 | 2,4499799501 |
| ENSG00000275385.1 | CCL18 | 2,4239296613 |
| ENSG00000281527.1 | AC087350.1 | 2,4138869313 |
| ENSG00000131370.14 | SH3BP5 | 2,4008012907 |
| ENSG00000102970.9 | CCL17 | 2,3892299866 |
| ENSG00000148541.11 | FAM13C | 2,3775039103 |
| ENSG00000141433.11 | ADCYAP1 | 2,3738123616 |
| ENSG00000166004.13 | CEP295 | 2,3710503862 |
| ENSG00000255423.1 | EBLN2 | 2,351485369 |
| ENSG00000172197.10 | MBOAT1 | 2,348166417 |
| ENSG00000198203.8 | SULT1C2 | 2,3463920493 |
| ENSG00000173213.8 | RP11-683L23.1 | 2,2985266278 |
| ENSG00000140650.10 | PMM2 | 2,2954829451 |
| ENSG00000165300.7 | SLITRK5 | 2,2902399888 |
| ENSG00000278888.1 | AC090154.1 | 2,2884424557 |
| ENSG00000111886.10 | GABRR2 | 2,2867048393 |
| ENSG00000169629.10 | RGPD8 | 2,2748980373 |
| ENSG00000089505.16 | CMTM1 | 2,270918422 |
| ENSG00000176358.14 | TAC4 | 2,2669354224 |
| ENSG00000160200.16 | CBS | 2,262604657 |
| ENSG00000136352.16 | NKX2-1 | 2,259262661 |
| ENSG00000243232.4 | PCDHAC2 | 2,2484649339 |
| ENSG00000136925.13 | TSTD2 | 2,2467939439 |
| ENSG00000204410.13 | MSH5 | 2,2205106861 |
| ENSG00000111012.8 | CYP27B1 | 2,2204876586 |
| ENSG00000127074.13 | RGS13 | 2,2175548256 |
| ENSG00000130684.12 | ZNF337 | 2,2042856738 |
| ENSG00000015479.16 | MATR3 | 2,175899316 |
| ENSG00000105492.14 | SIGLEC6 | 2,1540277149 |
| ENSG00000174482.9 | LINGO2 | 2,1496432845 |
| ENSG00000257315.1 | ZBED6 | 2,131631249 |
| ENSG00000111536.4 | IL26 | 2,1297869514 |
| ENSG00000172752.13 | COL6A5 | 2,1276494628 |
| ENSG00000147614.3 | ATP6V0D2 | 2,127548487 |
| ENSG00000161958.9 | FGF11 | 2,1260603165 |
| ENSG00000257446.3 | ZNF878 | 2,1105451328 |
| ENSG00000087303.15 | NID2 | 2,1065996781 |
| ENSG00000144063.3 | MALL | 2,1064878087 |
| ENSG00000243708.7 | PLA2G4B | 2,1053536887 |
| ENSG00000188257.9 | PLA2G2A | 2,0767904692 |
| ENSG00000241404.5 | EGFL8 | 2,0712920803 |
| ENSG00000133048.11 | CHI3L1 | 2,0700810374 |
| ENSG00000103226.16 | NOMO3 | 2,0695127715 |
| ENSG00000151967.17 | SCHIP1 | 2,0672758246 |
| ENSG00000133063.14 | CHIT1 | 2,0576537483 |
| ENSG00000165474.5 | GJB2 | 2,0424305624 |
| ENSG00000137976.7 | DNASE2B | 2,0392313138 |
| ENSG00000186714.11 | CCDC73 | 2,0386011299 |
| ENSG00000127366.5 | TAS2R5 | 2,0375979912 |
| ENSG00000123496.6 | IL13RA2 | 2,0325412173 |
| ENSG00000141668.8 | CBLN2 | 2,031991943 |
| ENSG00000165837.10 | ERICH6B | 2,0261172571 |
| ENSG00000196329.9 | GIMAP5 | 2,0168421019 |
| ENSG00000178175.10 | ZNF366 | 2,0037922445 |
| **Genes Down-regulated in FAT1 mutated vs. FAT1 wild-type cases** | | |
| ENSG00000144908.12 | ALDH1L1 | -2,0046585184 |
| ENSG00000253293.4 | HOXA10 | -2,005232369 |
| ENSG00000181240.12 | SLC25A41 | -2,0392488871 |
| ENSG00000122367.18 | LDB3 | -2,0404516333 |
| ENSG00000187134.11 | AKR1C1 | -2,0440224857 |
| ENSG00000151632.15 | AKR1C2 | -2,0502132989 |
| ENSG00000133636.9 | NTS | -2,0652422848 |
| ENSG00000135917.12 | SLC19A3 | -2,0666065065 |
| ENSG00000196616.11 | ADH1B | -2,0670192761 |
| ENSG00000087250.7 | MT3 | -2,074220955 |
| ENSG00000102468.9 | HTR2A | -2,0773690638 |
| ENSG00000135447.15 | PPP1R1A | -2,0854448228 |
| ENSG00000164920.8 | OSR2 | -2,0930436929 |
| ENSG00000188730.4 | VWC2 | -2,0975135911 |
| ENSG00000227471.7 | AKR1B15 | -2,1067762423 |
| ENSG00000153446.14 | C16orf89 | -2,1220263218 |
| ENSG00000161281.9 | COX7A1 | -2,1533405087 |
| ENSG00000154493.16 | C10orf90 | -2,1543356857 |
| ENSG00000198883.10 | PNMA5 | -2,1618584397 |
| ENSG00000078399.14 | HOXA9 | -2,1667791425 |
| ENSG00000070031.3 | SCT | -2,1687248724 |
| ENSG00000048540.13 | LMO3 | -2,1905849043 |
| ENSG00000243444.6 | PALM2 | -2,2016982194 |
| ENSG00000203867.7 | RBM20 | -2,2060239126 |
| ENSG00000149596.6 | JPH2 | -2,2119109077 |
| ENSG00000174429.3 | ABRA | -2,2119534834 |
| ENSG00000174899.9 | PQLC2L | -2,2264721564 |
| ENSG00000135346.7 | CGA | -2,236358116 |
| ENSG00000114200.8 | BCHE | -2,2384975211 |
| ENSG00000277117.3 | ICOSLG | -2,2497997615 |
| ENSG00000186367.6 | KIAA1024L | -2,2610533058 |
| ENSG00000110786.16 | PTPN5 | -2,2676468383 |
| ENSG00000101335.8 | MYL9 | -2,271855358 |
| ENSG00000164729.7 | SLC35G3 | -2,2791699309 |
| ENSG00000004776.10 | HSPB6 | -2,2849904931 |
| ENSG00000163431.12 | LMOD1 | -2,2937795571 |
| ENSG00000185352.8 | HS6ST3 | -2,2971203304 |
| ENSG00000005073.5 | HOXA11 | -2,3021824914 |
| ENSG00000197406.7 | DIO3 | -2,3339648709 |
| ENSG00000158497.2 | HMHB1 | -2,3391282452 |
| ENSG00000274443.3 | C8orf89 | -2,353124109 |
| ENSG00000160221.15 | C21orf33 | -2,3582517899 |
| ENSG00000124102.4 | PI3 | -2,3643465636 |
| ENSG00000162409.9 | PRKAA2 | -2,3709844608 |
| ENSG00000060709.12 | RIMBP2 | -2,374733075 |
| ENSG00000163395.15 | IGFN1 | -2,3811695094 |
| ENSG00000163017.12 | ACTG2 | -2,3850156119 |
| ENSG00000204335.3 | SP5 | -2,3940293324 |
| ENSG00000167676.3 | PLIN4 | -2,4181041199 |
| ENSG00000180818.4 | HOXC10 | -2,4193261405 |
| ENSG00000163273.3 | NPPC | -2,4377146029 |
| ENSG00000162594.13 | IL23R | -2,4489577828 |
| ENSG00000130822.14 | PNCK | -2,4625751077 |
| ENSG00000167641.9 | PPP1R14A | -2,4631175798 |
| ENSG00000184601.9 | C14orf180 | -2,469951698 |
| ENSG00000184486.8 | POU3F2 | -2,4733737297 |
| ENSG00000100867.13 | DHRS2 | -2,4802339248 |
| ENSG00000146267.11 | FAXC | -2,4812109496 |
| ENSG00000275895.3 | U2AF1 | -2,4892556398 |
| ENSG00000241935.7 | HOGA1 | -2,4923621069 |
| ENSG00000101443.16 | WFDC2 | -2,4927272155 |
| ENSG00000198889.4 | DCAF12L1 | -2,5311323342 |
| ENSG00000018625.13 | ATP1A2 | -2,5349096092 |
| ENSG00000167414.4 | GNG8 | -2,5723987293 |
| ENSG00000141744.3 | PNMT | -2,5741090627 |
| ENSG00000004846.15 | ABCB5 | -2,5751681737 |
| ENSG00000187288.9 | CIDEC | -2,6230409031 |
| ENSG00000166183.14 | ASPG | -2,6285877821 |
| ENSG00000166049.10 | PASD1 | -2,6377273575 |
| ENSG00000165323.14 | FAT3 | -2,6469994087 |
| ENSG00000158246.7 | FAM46B | -2,6754798723 |
| ENSG00000184811.3 | TUSC5 | -2,6801054317 |
| ENSG00000101251.10 | SEL1L2 | -2,7095576602 |
| ENSG00000126950.7 | TMEM35 | -2,7547763301 |
| ENSG00000173406.14 | DAB1 | -2,8157392142 |
| ENSG00000104941.6 | RSPH6A | -2,9774199124 |
| ENSG00000064205.9 | WISP2 | -2,986553323 |
| ENSG00000102230.12 | PCYT1B | -3,0032470712 |
| ENSG00000109846.6 | CRYAB | -3,0082718016 |
| ENSG00000172137.17 | CALB2 | -3,0312194269 |
| ENSG00000160180.15 | TFF3 | -3,0524915345 |
| ENSG00000141639.10 | MAPK4 | -3,0752615687 |
| ENSG00000204644.8 | ZFP57 | -3,0809948041 |
| ENSG00000141052.16 | MYOCD | -3,1360681625 |
| ENSG00000134184.11 | GSTM1 | -3,1556637047 |
| ENSG00000204334.7 | ERICH2 | -3,1647791303 |
| ENSG00000165186.10 | PTCHD1 | -3,1724593182 |
| ENSG00000175084.10 | DES | -3,181093799 |
| ENSG00000130176.6 | CNN1 | -3,1927834214 |
| ENSG00000173641.16 | HSPB7 | -3,2425461634 |
| ENSG00000173432.9 | SAA1 | -3,2890574682 |
| ENSG00000186439.11 | TRDN | -3,3395026271 |
| ENSG00000000005.5 | TNMD | -3,4688358713 |
| ENSG00000154620.5 | TMSB4Y | -3,6151785735 |
| ENSG00000187010.17 | RHD | -3,669479269 |
| ENSG00000183878.14 | UTY | -3,7505207091 |
| ENSG00000075891.20 | PAX2 | -3,7717975388 |
| ENSG00000165246.11 | NLGN4Y | -3,8007747152 |
| ENSG00000120251.17 | GRIA2 | -3,8024077619 |
| ENSG00000183036.9 | PCP4 | -3,8343463774 |
| ENSG00000267978.4 | MAGEA9 | -3,8423089216 |
| ENSG00000166796.10 | LDHC | -4,0148955573 |
| ENSG00000174697.4 | LEP | -4,0267119263 |
| ENSG00000067646.10 | ZFY | -4,1723666961 |
| ENSG00000114374.11 | USP9Y | -4,5107754507 |
| ENSG00000067048.15 | DDX3Y | -4,7493460045 |
| ENSG00000198692.8 | EIF1AY | -4,8212638504 |
| ENSG00000012817.14 | KDM5D | -4,9603390209 |
| ENSG00000129824.14 | RPS4Y1 | -5,5967993805 |

**Supplementary Figures**

**Fig. S1:** Unsupervised analysis (Principal Component Analysis) shows GATA3 and TBX21/T-bet sub-classification of Peripheral T-cell lymphomas not otherwise specified according to the signature reported by Iqbal et al. applied to the 21 cases of the discovery panel.


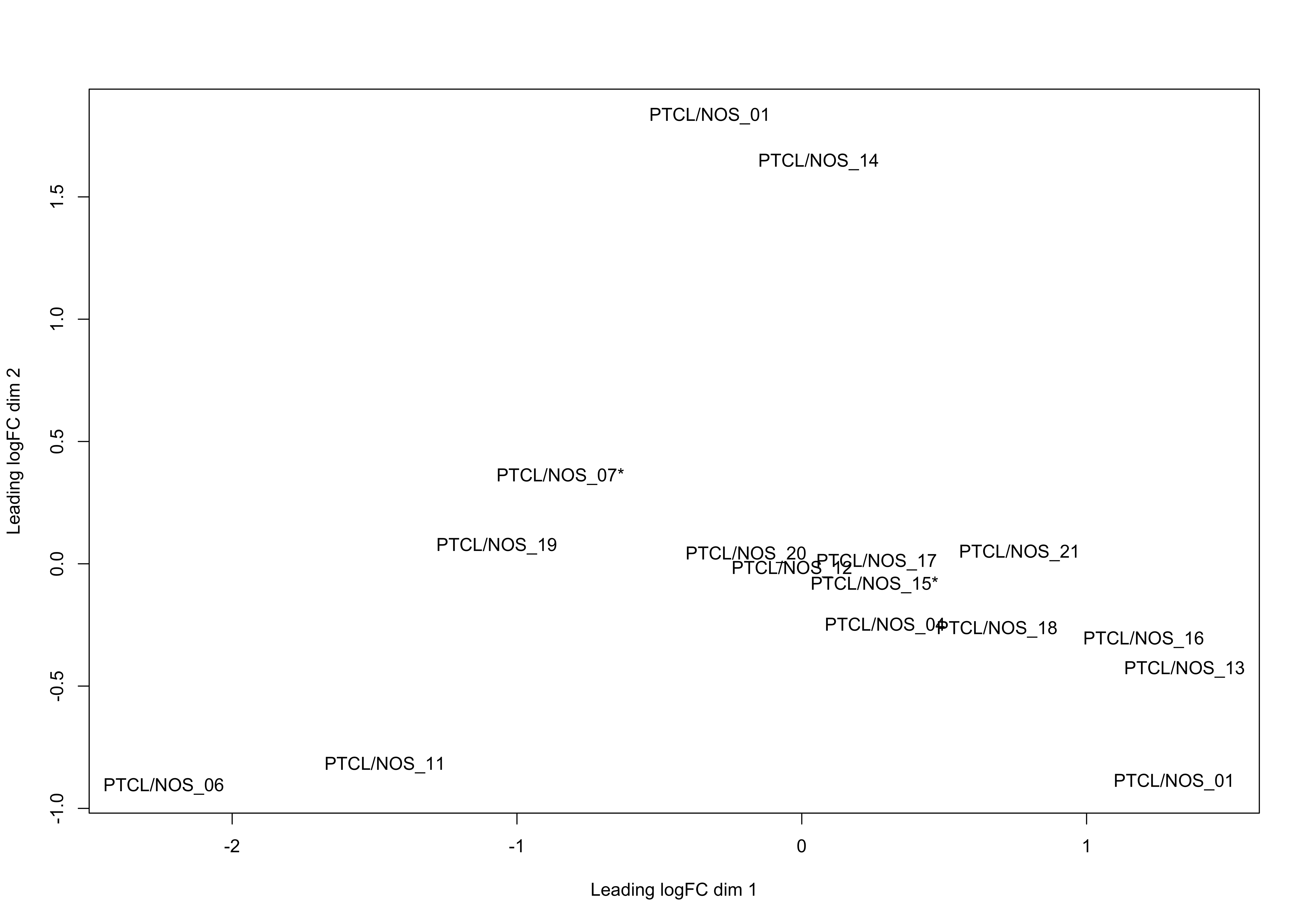


**Fig. S2:** Representative examples of TBX21/T-bet and GATA3 expression in Peripheral T-cell lymphoma not otherwise specified: TBX21/T-bet (a) and GATA3 (c) positive samples. Arrows highlight internal positive controls in TBX21/T-bet (b) and GATA3 (d) negative samples.


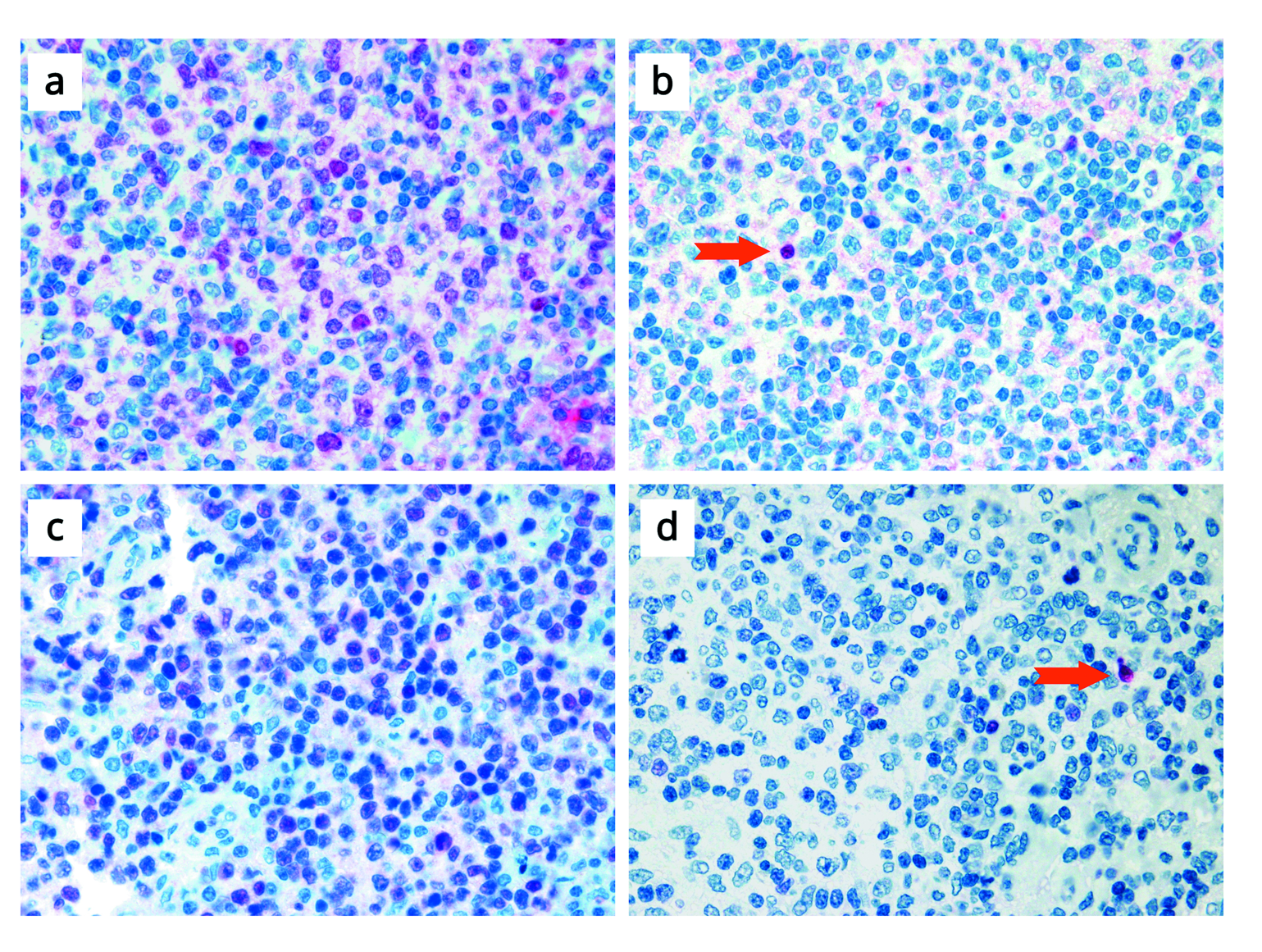


**Fig. S3:** Supervised analyses identified 209 differentially expressed genes comparing *FAT1* mutated *vs.* *FAT1* wild-type samples. In the matrix, each row represents a gene and each column represents a sample. The colour scale illustrates the relative expression level of a gene across all samples. Red bar= *FAT1* mutated samples, Blue bar= *FAT1* wild-type samples.


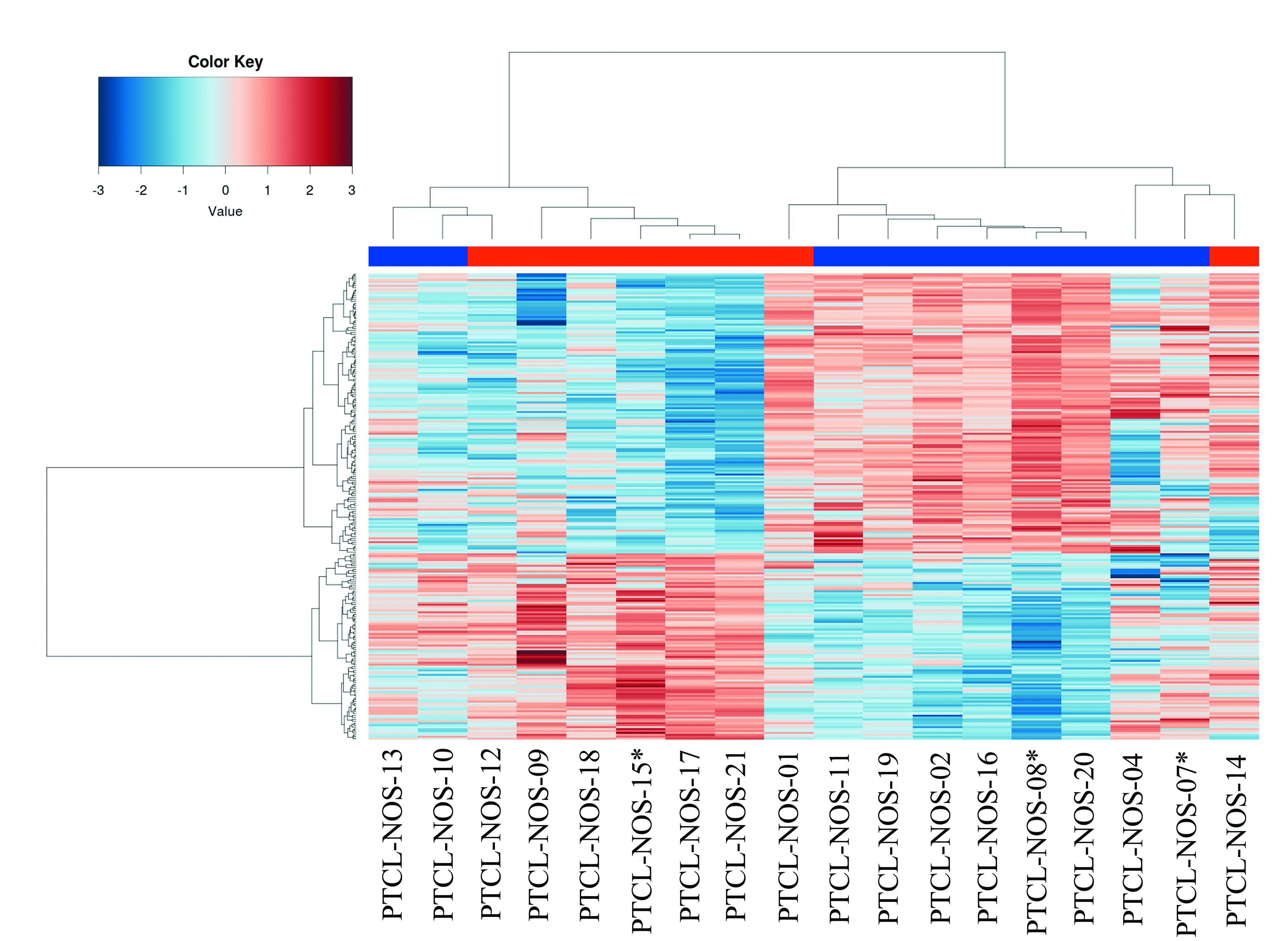


**Fig. S4:** The three GSEA revealed significant enrichment of genes up-regulated in *FAT1* mutated samples respect to *FAT1* wild-type samples. FAT1 mutated cases presented a signature enriched of genes involved in growth, apoptosis, cell migration, and invasiveness (FDR q-value ≤ 0.01). The enrichment score curve was obtained from GSEA software. In the enrichment plot, the x-axis shows the rank order of genes from the most upregulated to the most downregulated between *FAT1* mutated and *FAT1* wild-type samples. Vertical black line indicates the position of the enriched genes (Hit) comprising the gene set. The graph on the bottom shows the ranked list metric (signal-to-noise ratio) for each gene as a function of the rank in the ordered dataset.





**Fig. S5:** Survival analyses of samples mutated in chromatin remodeling genes (*KMT2C, KMT2D, KMT2A, SETD2* and *CHD1*) compared to wild-type samples. The limit of significance for the log-rank Mantle–Cox test was defined as P<0.05


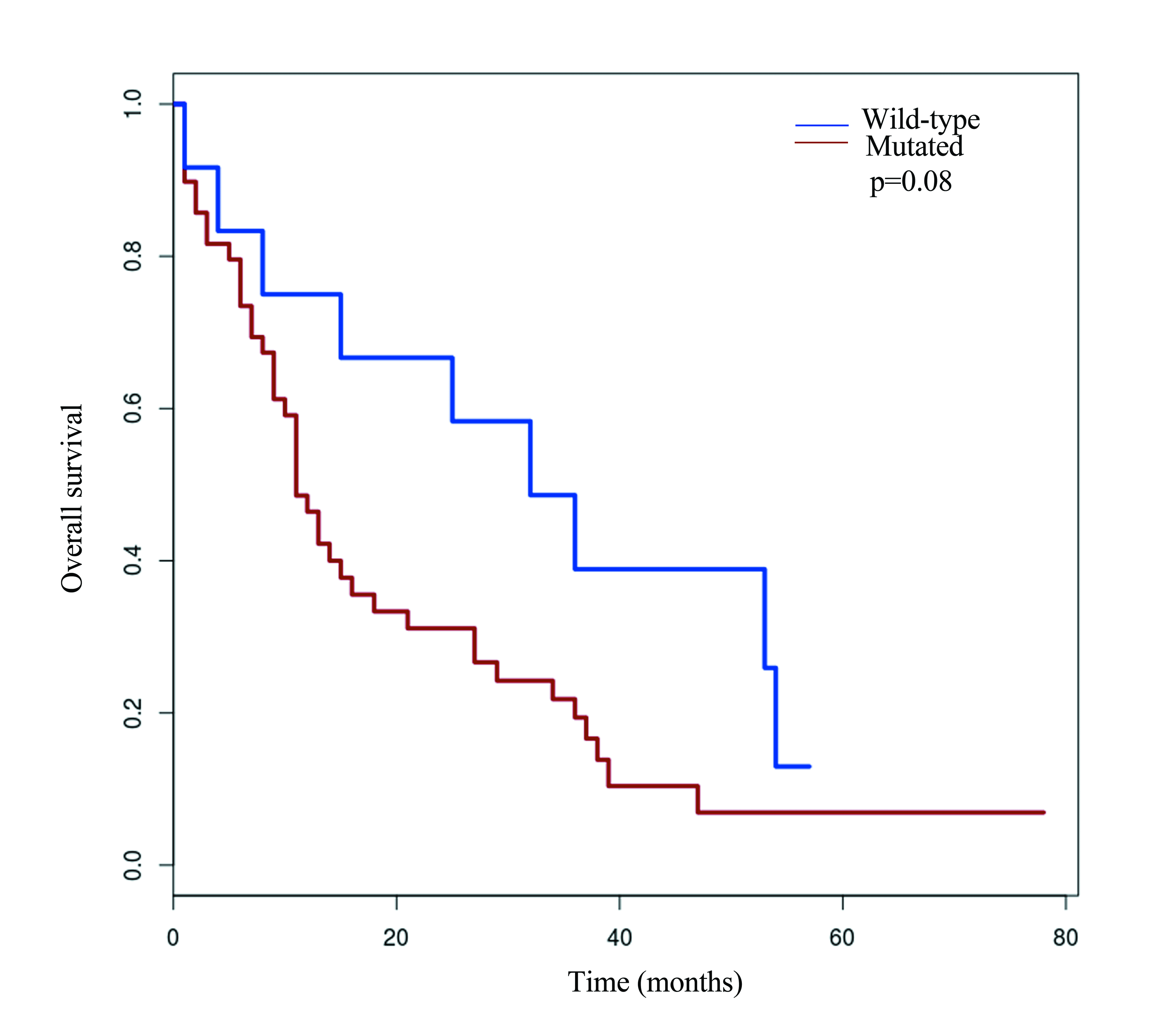

Supplement: Supplementary file 1 — Supplementary_File [file 41379_2019_279_MOESM1_ESM.docx]
